# Predicting treatment response using EEG in major depressive disorder: A machine-learning meta-analysis

**DOI:** 10.1038/s41398-022-02064-z

**Published:** 2022-08-12

**Authors:** Devon Watts, Rafaela Fernandes Pulice, Jim Reilly, Andre R. Brunoni, Flávio Kapczinski, Ives Cavalcante Passos

**Affiliations:** 1 Neuroscience Graduate Program, McMaster University, Hamilton, Canada; 2 School of Medicine, Universidade Federal Do Rio Grande Do Sul, Porto Alegre, RS Brasil; 3 Laboratório de Molecular Psychiatry, Centro de Pesquisa Experimental (CPE) and Centro de Pesquisa Clínica (CPC), Hospital de Clínicas de Porto Alegre (HCPA), Porto Alegre, RS Brasil; 4 Department of Electrical & Computer Engineering, McMaster University, Hamilton, ON Canada; 5 Service of Interdisciplinary Neuromodulation, Laboratory of Neurosciences (LIM-27), Institute of Psychiatry, University of São Paulo, São Paulo, Brasil; 6 Departamento de Clínica Médica, Faculdade de Medicina da USP, São Paulo, Brasil; 7 Instituto Nacional de Ciência e Tecnologia Translacional em Medicina (INCT-TM), Porto Alegre, RS Brasil; 8 Department of Psychiatry and Behavioural Neurosciences, McMaster University, Hamilton, ON Canada

**Keywords:** Predictive markers, Physiology

## Abstract

Selecting a course of treatment in psychiatry remains a trial-and-error process, and this long-standing clinical challenge has prompted an increased focus on predictive models of treatment response using machine learning techniques. Electroencephalography (EEG) represents a cost-effective and scalable potential measure to predict treatment response to major depressive disorder. We performed separate meta-analyses to determine the ability of models to distinguish between responders and non-responders using EEG across treatments, as well as a performed subgroup analysis of response to transcranial magnetic stimulation (rTMS), and antidepressants (Registration Number: CRD42021257477) in Major Depressive Disorder by searching PubMed, Scopus, and Web of Science for articles published between January 1960 and February 2022. We included 15 studies that predicted treatment responses among patients with major depressive disorder using machine-learning techniques. Within a random-effects model with a restricted maximum likelihood estimator comprising 758 patients, the pooled accuracy across studies was 83.93% (95% CI: 78.90–89.29), with an Area-Under-the-Curve (AUC) of 0.850 (95% CI: 0.747–0.890), and partial AUC of 0.779. The average sensitivity and specificity across models were 77.96% (95% CI: 60.05–88.70), and 84.60% (95% CI: 67.89–92.39), respectively. In a subgroup analysis, greater performance was observed in predicting response to rTMS (Pooled accuracy: 85.70% (95% CI: 77.45–94.83), Area-Under-the-Curve (AUC): 0.928, partial AUC: 0.844), relative to antidepressants (Pooled accuracy: 81.41% (95% CI: 77.45–94.83, AUC: 0.895, pAUC: 0.821). Furthermore, across all meta-analyses, the specificity (true negatives) of EEG models was greater than the sensitivity (true positives), suggesting that EEG models thus far better identify non-responders than responders to treatment in MDD. Studies varied widely in important features across models, although relevant features included absolute and relative power in frontal and temporal electrodes, measures of connectivity, and asymmetry across hemispheres. Predictive models of treatment response using EEG hold promise in major depressive disorder, although there is a need for prospective model validation in independent datasets, and a greater emphasis on replicating physiological markers. Crucially, standardization in cut-off values and clinical scales for defining clinical response and non-response will aid in the reproducibility of findings and the clinical utility of predictive models. Furthermore, several models thus far have used data from open-label trials with small sample sizes and evaluated performance in the absence of training and testing sets, which increases the risk of statistical overfitting. Large consortium studies are required to establish predictive signatures of treatment response using EEG, and better elucidate the replicability of specific markers. Additionally, it is speculated that greater performance was observed in rTMS models, since EEG is assessing neural networks more likely to be directly targeted by rTMS, comprising electrical activity primarily near the surface of the cortex. Prospectively, there is a need for models that examine the comparative effectiveness of multiple treatments across the same patients. However, this will require a thoughtful consideration towards cumulative treatment effects, and whether washout periods between treatments should be utilised. Regardless, longitudinal cross-over trials comparing multiple treatments across the same group of patients will be an important prerequisite step to both facilitate precision psychiatry and identify generalizable physiological predictors of response between and across treatment options.

## Introduction

It has been notably demonstrated in the Sequential Treatment Alternatives to Relieve Depression (STAR*D) study that antidepressants fail to facilitate remission in most patients with major depressive disorder (MDD) and that there is no clearly preferred medication when patients inadequately respond to several courses of antidepressants [1]. Similarly, data from a multicentre randomized controlled trial spanning 2439 patients across 73 general practices in the UK found that 55% of patients (95% CI: 53–58%) met the threshold for treatment-resistant depression, defined as ≥14 on the BDI-II, and who had been taking antidepressant medication of an adequate dose, for at least 6 weeks [2].

This long-standing clinical challenge of selecting an appropriate treatment for any given patient has prompted the increasing development of predictive models of treatment response using machine learning techniques. Broadly speaking, supervised machine learning models use labeled training data (e.g., features or input variables), to predict a given outcome (e.g., treatment response) in unseen data (e.g., testing or validation dataset) [3]. In the context of psychiatry, these models have largely involved classification and regression tasks, where the outcome is a categorical (e.g., responders vs. non-responders), or a continuous outcome (e.g., depression change scores). There are several available algorithms to select from, each relying on a series of assumptions of the underlying input data. Moreover, an important consideration in model development is hyperparameter tuning, which involves finding a configuration of tuning parameters prior to model training that results in the best performance (e.g., accuracy for classification models, and lowest root mean squared error for regression models, respectively). A detailed overview of supervised machine learning [4], algorithm selection [3], and hyperparameter tuning [5] can be found elsewhere.

Thus far, most studies have utilized baseline clinical data to predict prospective treatment response at an individual level, with varying degrees of success and methodological robustness [6]. Similarly, there is a growing interest in the use of neuroimaging and neurophysiological markers as input features to these models. For instance, in a recent meta-analysis using MRI to predict treatment response in MDD, comprising 957 patients, the overall area under the bivariate summary receiver operating curve (AUC) was 0.84, with no significant difference in performance between treatments or MRI machines [7]. AUC, as described elsewhere [8], is a measure ranging from 0 to 1 indicating how well a parameter can distinguish between two diagnostic groups (e.g., responders/non-responders to an intervention).

However, fMRI and MRI remain impractical as widespread clinical tools to predict treatment response in psychiatry, considering the high costs associated with each scan, and the excessive wait times to access a limited number of MRI machines. It was also recently shown in a landmark study that due to considerable analytical flexibility in fMRI pipelines, seventy independent teams yielded notably different conclusions when presented with the same dataset and series of hypotheses [9].

In contrast, measures such as electroencephalography (EEG) are comparably more cost-effective and scalable as a potential clinical tool to predict treatment response. As described elsewhere [10], EEG oscillations refer to rhythmic electrical activity in the brain and constitute a mechanism where the brain can regulate changes within selected neuronal networks. This repetitive brain activity emerges because of the interactions of large populations of neurons. As such, there is evidence that MDD may be related to abnormalities in large-scale cortical and subcortical systems distributed across frontal, temporal, parietal, and occipital regions [10].

For instance, power amplitudes in specific frequency bands, known as band power, are associated with different mechanisms in the brain. Although incompletely understood, alpha band power (8–12 Hz) reflects sensory and attentional inhibition and has been shown to be associated with creative ideation [11], beta frequencies (13–30 Hz) are prominent during problem-solving [12, 13], while delta frequencies (≤4 Hz) are notable during deep sleep [14], gamma frequencies (30–80 Hz) during intensive concentration [15], and greater theta band frequencies (4–8 Hz) during relaxation, respectively [16]. Alpha asymmetry, which measures the relative alpha band power between hemispheres, particularly within frontal electrodes, has been shown to discriminate individuals with MDD from healthy controls, although inconsistencies have been found across literature [17]. Similarly, beta and low gamma powers in fronto-central regions have been shown to be negatively correlated with inattention scores in MDD [18]. Moreover, intrinsic local beta oscillations in the subgenual cingulate were found to be inversely related to depressive symptoms, particularly in the lower beta range of ~13–25 Hz [19]. Additionally, in specific contexts, gamma rhythms, which represent neural oscillations between 25 and 140 Hz, have been shown to distinguish patients with MDD from healthy controls, and various therapeutic agents for depression have also been shown to alter gamma oscillations [20]. Patients with depression also show more random network structure, and differences in signal complexity [17], which may serve as replicable biomarkers of treatment response and remission.

A detailed description of potential EEG biomarkers of depression including signal features, evoked potentials, and transitions in resting-state EEG between wake and deep sleep, can be found elsewhere [17]. Altogether, no robust individual biomarker of treatment response in MDD has emerged. Towards this end, in a meta-analysis of treatment response prediction during a depressive episode, it was shown that the sensitivity across articles was 0.72 (95% CI = 0.67–0.76), and specificity was 0.68 (95% CI = 0.63–0.73), respectively [21]. Nonetheless, most included studies used linear discriminant analysis in the absence of adequate cross-validation methods, training, and testing sets, or hyperparameter tuning, which may have led to biased performance metrics and a greater likelihood of statistical overfitting. Therefore, in the present study, we aimed to meta-analyze and systematically review studies that used machine learning techniques to predict treatment response in MDD.

## Methods

This study has been registered on PROSPERO with the registration number PROSPERO CRD42021257477.

### Search strategy

Three electronic databases (PubMed, Scopus, and Web of Science) were examined for articles published between January 1960 and February 2022. To identify relevant studies, the following structure for the search terms was used: (Supervised Machine Learning OR Artificial Intelligence) AND (Major Depressive Disorder) AND (Electroencephalography) AND (Interventions OR Trials). The complete filter is available in the supplementary material. We also screened references from the included articles to identify potential missed articles. There were no language restrictions.

### Eligibility criteria

This meta-analysis was performed according to the PRISMA statement [22]. We selected original articles that assessed patients with a psychiatric disorder treated with pharmacological or non-pharmacological interventions coupled with machine learning models and electroencephalography (EEG) feature to predict treatment outcomes. Review articles and preclinical trials were excluded. A minimum criterion of cross-validation or training and testing sets were required for study inclusion since models lacking resampling procedures are less likely to appropriately generalize to independent datasets. Furthermore, studies with small sample sizes (≤30) that did not correct for overfitting were excluded, since cross-validation with small sample sizes, in the absence of training and testing sets, can lead to inflated and highly variable predictive accuracy [23]. Details relating to excluded studies can be found in Supplementary Table 1.

### Data collection and extraction

Initially, the potential articles were independently screened for title and abstract contents by two researchers (DW and RFP). Then, they also obtained and read the full text of potential articles. A third author (ICP) provided a final decision in cases of disagreement. Data extracted from the studies included publication year, sample size, diagnosis, EEG system, reference choice, impedance, number and type of electrodes, a method for de-artificing, feature selection and extraction method, type of intervention, outcomes of interest, machine learning algorithm, and performance metrics of the models (i.e., accuracy, balanced accuracy, sensitivity, specificity, area under the curve, true positive, false positive, true negative and false negative, and coefficient of determination). We also developed a quality assessment instrument specific to machine learning studies since there is no tool for quality assessment in machine learning studies. Briefly, the quality assessment evaluates studies according to several domains including representativeness of the sample, confounding variables, outcome assessment, machine learning approach, feature selection, class imbalance, missing data, performance/accuracy, and testing/validation. This instrument, and a brief description of each component, are further described in the Supplementary Material. Additionally, we utilized the Quality Assessment of Diagnostic Accuracy Studies-2 (QUADAS-2) [24] to assess potential bias and variation in each included study, as described in Supplementary Table 2.

In terms of the analysis, “mada” [25], “dmetatools” and “meta” packages in R were used to meta-analyze diagnostic accuracy studies. The metamean function in the “meta” package was used to pool accuracy across studies in a random-effects model using an inverse variance method with Knapp–Hartung adjustments to calculate the confidence interval around the pooled effect. A restricted maximum-likelihood estimator was used to calculate the heterogeneity variance *τ*^2^. Moreover, the madad function in the “mada” package was used to calculate the sensitivity, specificity, and pAUC across studies, while the Madauni function was used to calculate the Diagnostic Odds Ratio (DOR), positive likelihood ratio (posLR), and negative likelihood ratio (negLR). AUC was calculated using the AUC_boot function in dmetatools, with an alpha of 0.95 and 2000 bootstrap iterations.

## Results

We found 2489 potential abstracts and included 15 articles in the present meta-analysis and systematic review, two included after reference screening (Supplementary Table). A list of included studies as well as their most relevant characteristics and findings are detailed in Table 1. Two separate quality assessments can be observed in the supplementary material. Of the included studies, seven predicted responses to brain stimulation therapies [26–30, 32, 33], and eight predicted responses to pharmacological treatment [34–41]. Additionally, a complete breakdown of how each study defined treatment response can be found in Supplementary Table S4.

**Table 1:** Machine learning studies predicting treatment response using EEG in major depressive disorder (a summary of sample size, treatment outcomes, machine learning algorithms, and performance metrics).

First author, year	Sample size and diagnosis [1, 2]	Intervention	Outcome	Machine learning model	Accuracy	Other measures
*Studies predicting response to neurostimulation therapy*						
Bailey [26]	39 patients with treatment-resistant depression	3 weeks (15 sessions) unilateral left 10 Hz rTMS	Responders (≥50% decrease in HAM-D after 5–8 weeks of rTMS) vs. Non-responders	Linear SVM	91%	Sensitivity: 91% Specificity: 92% F1 score: 0.93
Bailey [26]	32 patients with treatment-resistant depression	3 weeks (15 sessions) unilateral left 10 Hz rTMS	Responders (≥50% decrease in HAM-D after 5–8 weeks of rTMS) vs. Non-responders	Linear SVM	86.66%	Sensitivity: 84% Specificity: 89%
Corlier [28]	109 patients with MDD	3 weeks (15 sessions) of 10 Hz left DLPFC rTMS (68 subjects received unilateral left treatment, 41 were changed to sequential bilateral treatment—10 Hz left DLPFC, 1 Hz right DLPFC)	Responders (≥40% decrease in IDS-30 scores from baseline to treatment 30) vs. Non-responders	Elastic Net	61.8–79.2% (Best performance observed with alpha band frequency and IDS-30 percent change score)	AUC: 0.52–0.77 Specificity: 70.9–82.7% Sensitivity: 34.8–75.7% PPV: 58.2–79.7% NPV: 63.8–82.2%
Erguzel [29]	147 patients with treatment-resistant depression	18 sessions of 25 Hz left PFC rTMS	Responders (≥50% decrease in HAM-D scores after 3 weeks of treatment) vs. Non-responders	BPNN	89.12%	Sensitivity: 94.44% AUC: 0.904
Erguzel [30]	55 patients with MDD	18 sessions of 25 Hz left PFC rTMS	Responders (≥50% decrease in HAM-D scores after 3 weeks of treatment) vs. Non-responders	ANN	89.09%	Sensitivity: 86.67–93.33% Specificity: 80–84% AUC: 0.686–0.909 Best model (6-fold CV) Sensitivity: 93.3% Specificity: 84.0% AUC: 0.909
Erguzel [31]	147 patients with treatment-resistant depression	20 sessions of adjunctive 25 Hz left PFC rTMS	Responders (≥50% decrease in HAM-D scores after 20 sessions of rTMS) vs. Non-responders	ANN SVM DT	Accuracy: 78.3–86.4% Best performance using SVM Balanced Accuracy: 54.71–75.42%	Sensitivity: 60.41–68.62% Specificity: 49.01–82.22%
Hasanzadeh [33]	46 patients with MDD	5-sessions of 10 Hz left DLPFC rTMS	Responders (≥50% decrease in BDI-II or HAM-D scores from baseline) vs. Non-responders Remission (Remission defined as BDI ≤ 8 or HAM-D ≤ 9) vs. Non-remission	kNN	76.1–91.3% best performance with power spectral features	Sensitivity: 69.6–87% Specificity: 82.6–95.7%
*Studies predicting response to pharmacological treatment*						
Cao [34]	37 patients with treatment-resistant depression	Patients randomized to one of three groups (1:1:1): 0.5 mg/kg ketamine 0.2 mg/kg ketamine Normal saline	Responders (≥45% reduction in HAM-D score from baseline to 240 min posttreatment) vs. Non-responders	LDA NMSC kNN PARZEN PERLC DRBMC SVM Radial kernel	78.4% Best performance using SVM with a radial kernel	Sensitivity: 79.3% Specificity: 84.2% Recall: 78.5% Precision: 87.0% *F*1 score: 52.6%
Cook [35]	180 patients with MDD	8-week trial of escitalopram (10 mg) or bupropion (150 mg) (1-week single-blind escitalopram followed by 7 weeks double-blind trial)	Remission (≤7 HDRS at week 8) vs. Non-remission	LDA	64.4%	Sensitivity: 74.3% Specificity: 55.3% PPV: 60.5% NPV: 70.0% AUC: 0.635
de la Salle [36]	47 patients with MDD	12-week double-blinded trial of: (1) escitalopram+ bupropion (2) escitalopram+ placebo (3) bupropion+placebo	Responders (≥50% reduction in MADRS scores from baseline to posttreatment) vs. Non-responders Remitters (≤10 MADRS at post-treatment) vs. Non-responders	LR	Response: Change in PF Cordance: 81% Change in MRF Cordance: 74% Remission: Change in PF Cordance: 70% Change in MRF Cordance: 51%	Response (ΔPF): AUC: 0.85 Sensitivity: 70% Specificity: 85% PPV: 0.95 NPV: 0.76 Remission (ΔPF): AUC: 0.66 Sensitivity: 65% Specificity: 74% PPV: 65% NPV: 74% Response (ΔMRF): AUC: 0.80 Sensitivity: 70% Specificity: 95% PPV: 95% NPV: 76% Remission (ΔMRF): AUC: 0.59 Sensitivity: 93% Specificity: 31% PPV: 39% NPV: 91%
Jaworska [37]	51 patients with MDD	12-week double-blinded trial of: (1) escitalopram+bupropion (2) escitalopram+placebo (3) bupropion+placebo	Responders (≥50% reduction in MADRS scores from baseline to posttreatment) vs. Non-responders	RF SVM AdaBoost CART MLP GNB	88% Combined model, accuracy of each individual model not reported	AUC: 0.716-0.901 Highest AUC observed in Random Forest Model Combined model Sensitivity = 77% Specificity = 99% PPV = 99 NPV = 81
Mumtaz [38]	34 patients with MDD	Open-label trial of an SSRI	Responders (Responders defined as ≥50% improvement in pre- vs. post-treatment BDI-II scores) vs. Non-responders	LR	87.5%	Sensitivity: 95% Specificity: 80%
Rajpurkar [39]	518 patients with MDD	Patients randomized in a 1:1:1: ratio to escitalopram, sertraline, or extended-release venlafaxine for 8 weeks	Regression model (Continuous improvement in individual symptoms, defined as the difference in score for each of the symptoms on the HAM-D from baseline to week 8)	GBM	*R*^2^ = 0.375–0.551 Best model observed using EEG and baseline symptom features	95% CI: 0.473–0.639 Used C-index to assess performance (probability that the algorithm will correctly identify, given 2 random patients with different improvement levels, which patient showed greater improvement
Wu [40]	309 patients with MDD	8-week course of sertraline or placebo	Regression model (Pre- minus post-treatment difference in HAMD17 scores, with missing endpoint values, imputed to maintain an intent-to-treat framework.)	SELSER Algorithm developed in the current study	*R*^2^ = 0.60 Sertraline *R*^2^ = 0.41 Placebo	NA
Zhdanov [41]	122 patients with MDD	8-weeks of open-label escitalopram (10–20 mg) treatment	Responders (≥50% improvement in MADRS scores from baseline to post-treatment) vs. Non-responders	SVM radial kernel	79.2% Using baseline EEG data 82.4% Using baseline and week 2 EEG data	Baseline Model Sensitivity—67.3% Specificity—91.0% Baseline and Week 2 Model Sensitivity: 79.2% Specificity: 85.5%

*ANN* artificial neural network, *BDI* Beck depression inventory, *BPNN* back-propagation neural networks, *CART* classification and regression trees, *CNN* convolutional neural network, DLPFC dorsolateral prefrontal cortex, DRBMC discriminative restricted Boltzmann machine, *DT* decision trees, *ELM* extreme learning machine, *GBM* gradient boosting machine, *GNB* Gaussian naive Bayes, *HAM-D* Hamilton depression rating scale, *IDS-SR* inventory of depressive symptomatology (self-report), *kNN* *k*-nearest neighbors, *KPLSR* kernelized partial least squares regression, *LASSO* least absolute shrinkage and selection operator, *LDA* linear discriminant analysis, *LR* logistic regression; *MADRS* Montgomery–Asberg depression rating scale, *MFA* mixture of factor analysis, *MLP* multi-layer perceptron, *MRF* middle right frontal, *NMSC* nearest mean classifier, *PARZEN* Parzen density estimation, *PERCL* perceptron classifier, *RF* random forest, *SCZ* schizophrenia, *SELSER* sparse EEG latent SpacE regression, *SVM* support vector machine.

### Studies predicting treatment response to brain stimulation therapies

There were seven studies using EEG features to predict treatment response to brain stimulation [26–30, 32, 33]. Among these, all predicted responses to repetitive transcranial magnetic stimulation (rTMS). Further information relating to feature extraction methods, feature selection, and extracted features can be found in Table 2.

**Table 2:** Extracted features across studies (a summary of pre-processing strategies, feature extraction methods, feature selection, and top predictors across studies).

First author, year	Pre-processing strategy	EEG features	Feature extraction method	Feature selection method	Top features *Top 10 features, if applicable*
*Studies predicting response to neurostimulation therapy*					
Bailey [26]	Data down-sampled to 1000 Hz Second order Butterworth filtering with bandpass from 1 to 80 Hz and a band-stop filter 47–53 Hz Fast ICA used to manually select and remove eye blinks, movements, and remaining muscle artifacts.	Power spectral analysis connectivity analysis	*Power spectral analysis* - Morlet Wavelet transform to calculate power in the upper alpha band (10–12.5 Hz), theta band (4–8 Hz), and gamma band (30–45 Hz) - Average power calculated across the entire retention period with each frequency band and averaged over trials *Connectivity analysis* - Hanning taper time–frequency transform to determine instantaneous phase values for complex Fourier-spectra from 4 to 45 Hz with a 1 Hz resolution across a 3-oscillation sliding time window - Weighted phase lagged index (wPLI) calculated between each electrode - wPLI provides a value between 0 and 1 for each electrode pair at each frequency and time point	Not applicable	*Statistically significant variables between responders and non-responders; authors did not report top features in the total model* - Greater theta power at Fz in responders vs. non-responders (*F*1 = 8.577, *p* = 0.006) - No significant differences for alpha or gamma power, or theta-gamma coupling - Responders showed a non-significant pattern of less gamma connectivity than non-responders at baseline (*p* = 0.523), and greater gamma connectivity at week 1 (*p* = 0.0836). - Responders showed significantly more theta connectivity across baseline and week 1, with both interhemispheric fronto-parietal coupling and frontal and parietal interhemispheric coupling (overall *p* = 0.003).
Bailey [26]	Same Procedure as Bailey [26]	Power spectral analysis Connectivity analysis Theta cordance analysis	*Power and connectivity analyses follow the same procedure as Bailey 2017* *Theta cordance analysis* - Absolute power values for each epoch 1–80 Hz underwent a multi-taper fast Fourier frequency transformation with a Hanning taper - Absolute power averaged across neighboring electrode pairs - Relative power in reattributed absolute theta band calculated by dividing power in theta band by total power from 1 to 80 Hz - Subtracted half-maximal values from normalized absolute and relative power in theta band, and summed together for each electrode *iAPF analysis* - Individualized alpha peak frequency averaged across F3, Fz, and F4 electrodes - Multitaper fast Fourier frequency transformation - Gaussian distribution with least-squared error fitted to electrodes in 6–14 Hz range - Peaks of distribution selected from each electrode and averaged	Not applicable	*Statistically significant variables between responders and non-responders; authors did not report top features in the total model* - Greater theta connectivity in responders vs. non-responders (*p* = 0.0216, FDR *p* = 0.030). Responders showed atypical, elevated theta connectivity, while non-responders showed typical theta connectivity, which was comparable to controls. - No main effect of theta cordance, frontal-midline theta power, or alpha power.
Corlier [28]	ICA-based FASTER algorithm Dominant alpha frequency peak determined for each subject (highest spectral peak within 7-13 Hz alpha range)	EEG functional connectivity measures (coherence, envelope correlation, and alpha band frequency)	*Functional connectivity measures* - Coherence: correlation of amplitude and phase - Envelope: correlation of amplitude - Alpha frequency band: similarity of the spectral waveform of the alpha band across regions	Elastic Net	Coherence & Envelope: Connections in the frontal to temporo-parietal nodes Alpha frequency band: Connections between the left frontal seeds (near stimulation site) and contralateral fronto-temporal locations EN models for coherence and envelope correlation showed a diffuse coupling pattern, while αSC showed a more focal connectivity.
Erguzel [30]	Manually selected artifact-free EEG data with a minimum split-half reliability ratio of 0.95 and minimum test-retest reliability ratio of 0.90. FFT	EEG cordance (combines absolute and relative EEG power, and negative discordance values)	*EEG cordance* - Normalized power across electrode sites and frequency bands - Maximum absolute and relative power of each frequency band is calculated to derive normalized absolute and relative power - Half-maximal value is subtracted, absolute/relative normalized power is summed.	Genetic algorithm - adaptive heuristic search algorithm was applied to features of all selected channels to reduce the number of dimensions	Fp1, Fp2, F7, F8, and F3 in the theta frequency band
Erguzel [29]	Band-pass filter with 0.15–30 Hz frequency FFT used to calculate absolute and relative power in each of two non-overlapping frequency bands (Delta—1–4 Hz, theta—4–8 Hz)	EEG cordance (combines absolute and relative EEG power, and negative discordance values)	*EEG cordance* - Normalized power across electrode sites and frequency bands - Maximum absolute and relative power of each frequency band is calculated to derive normalized absolute and relative power - Half-maximal value is subtracted, absolute/relative normalized power is summed.	ANN	NA
Erguzel [31]	Band-pass filter with 0.15–30 Hz frequency Manually selected artifact-free EEG data (at least 2 min) FFT	EEG cordance (combines absolute and relative EEG power, and negative discordance values)	EEG cordance analyses follow the same procedure as Erguzel 2014	Not applicable	*Feature set was composed of frequency bands for six frontal electrodes (Fp1, Fp2, F3, F4, F7 and F8)*
Hasanzadeh [33]	Sampling frequency 500 Hz Bandpass FIR filter (1–42 Hz) ICA to remove noisy data MARA to label noisy ICs Visually inspected to eliminate remaining artifacts	21 features in four categories (nonlinear, PSDl, spectral, and cordance)	*Nonlinear features* - LZC: Complexity measure of time series to estimate scholastic and chaotic behavior of time series - KFD: Algorithm for computing fractal dimension, a measure of self-similarity of a time series based on number of patterns repetitions *Power spectral density* - Delta (1–4 Hz)—Beta (12–30 Hz) by Welch method with a non-overlapped window, 500 samples in length - Average power computed for frequencies in each band *Spectrum features* - Method that quantifies the degree of phase coupling between components of a signal *Cordance* - measure of complexity of system based on chaos and time delay reconstruction theory	mRMR	- Nonlinear (LZC, KFD, CD)—80.4% accuracy - Power (D, T, A, B) - 91.3% accuracy - Spectrum (BispSL, Bisp2M, and BispEn in all bands)—84.8% accuracy - Cordance (Fr, Pre, Fr)—76.1% accuracy - All—87% accuracy
*Studies predicting response to pharmacological treatment*					
Cao [34]	Real-time artifact removal algorithm based on CCA, feature extraction, and a GMM used to improve signal quality	Power spectral analysis EEG Alpha Asymmetry EEG Theta Cordance	*Power spectral analysis* - 256-point FFT using Welch’s method - 10 min spans of data with 256-point moving window at 128-point overlap - Absolute and relative power of four prefrontal channels from delta (1–3.5 Hz), theta (4–7.5 Hz), lower alpha (8–10 Hz) and upper alpha (10.5–12 Hz) bands. *EEG alpha asymmetry* - mid-prefrontal (Fp1/Fp2) and mid-lateral (AF7/AF8) hemispheric asymmetry index to establish a relative measure of the difference in EEG (lower and upper) alpha power between the right and left forehead areas. *EEG theta cordance* - Combines information from both absolute and relative powers in the EEG theta band	*p*-value: measured using the Wilcoxon rank-sum test with a significant *p*-value < 0.05.	0.5 mg/kg dose - AF7 theta—*p* = 0.042 - Fp2 theta—*p* = 0.035 0.2 mg/kg dose - Fp1 theta—*p* = 0.038 - Fp2 theta—*p* = 0.042
Cooks [35]	Artifact-free epochs selected following rejection of muscle, electrocardiographic, and drowsiness artifacts.	Power spectral analysis ATR Relative combined theta and alpha power	*Power spectral analysis* - Calculated using consecutive two-second epochs of eyes-closed rest, by averaging values calculated separately for each channel in each epoch *Relative combined theta and alpha power* - Non-linear weighted combination of relative combined theta and alpha power (3–112 Hz), alpha1 power (8.5–12 Hz) and alpha2 absolute power (9–11.5 Hz)	Relative combined theta and alpha power was scaled to a range from 0 to 100; a cut-off score of ≥46.2 was selected	NA
Jaworska [37]	Bandpass filters 0.1–80 Hz 100 s of artifact-free data subjected to a FFT ln-transformed prior to analyses to ensure normality (Minimizes influence of extreme values)	eLORETA analysis Theta Cordance	*eLORETA analysis* - estimates neural activity as current density based on MNI-152 template, creating a low-resolution activation image *Theta cordance* - Values from prefrontal electrodes (Fp1, Fp2) at baseline and week 1	Tree-based feature selection kernel PCA	eLORETA features were most important, comprising 17 delta, 20 theta, 14 alpha^1^, 20 alpha^2^, and 17 beta EEG features. *Delta* Power at week 1 at T8 followed by power at Cp6 *Theta* Baseline power at Fp2 and week 1 power at Fc2 Alpha^1^ Baseline power at F7/8 Alpha^2^ Baseline power at P8 and week 1 power at O1 *Beta* Baseline power at T7 and week 21 power at Fz
Mumtaz [38]	Bandpass filters 0.1–70 Hz EEG data collected during 5 min eyes open, and 5 min eyes closed - 3-stimulus visual Oddball task used 50 Hz notch filter used to suppress power line noise	Wavelet coefficients in the delta and theta frequency range	*Wavelet coefficients* - involves a window function to capture both low and high-frequency components of the signal	Rank-based feature selection according to their relevance to class labels minimum redundancy and maximum relevance	*Top EEG features:* Fp2—delta frequency C3—theta frequency F7—delta frequency F3—delta frequency F7—theta frequency T4—theta frequency F8—theta frequency F4—delta frequency Fz—delta frequency F4—delta frequency C4—delta frequency F8—theta frequency T4—delta frequency P3—theta frequency
Rajpurkar [39]	Raw EEG signal was filtered using a band-pass filter with 0.15 - 30 Hz frequency prior to artifact removal FFT	Relative and absolute band power Frontal alpha asymmetry Occipital asymmetry Ratio of beta/alpha band power Ratio of theta/alpha band power	*Relative/absolute power as described above* Frontal alpha asymmetry - difference in alpha bandpower between O2 and O1 Occipital beta asymmetry - difference in beta bandpower between O2 and O1 ratio of beta/alpha and theta/alpha band power - Calculated for each electrode Feature selection: Decision tree weight in LightGBM	Gradient boosted feature selection	*Top EEG features*: 1. T7-T3 alpha absolute ratio 2. T7-T3 beta absolute ratio 3. F7 gamma relative 4. Fp2 delta relative 5. F3 alpha absolute 6. Fp2 theta absolute 7. P4 alpha absolute 8. T7-T3 beta relative ratio 9. F7 beta relative
Salle [36]	Data was filtered (0.1–30 Hz), ocular-corrected, and inspected for artifacts (voltages ±μV, faulty channels, drift) Minimum of 100 s of artifact-free data was required for participant inclusion	Theta Cordance (Prefrontal— Fp1, Fp2 MRF—Fz, Fp2, F4, F8)	*EEG theta cordance* Combines information from both absolute and relative powers in the EEG theta band	NA	*Top EEG features:* Change in prefrontal theta cordance (Fp1 + Fp2) = 81% accuracy Change in MRF theta cordance (Fz, Fp2, F4, F8) = 74% accuracy
Wu [40]	60 Hz AC line noise artifact removed using CleanLine - Non-physiological slow drifts in EEG recordings were removed using 0.01 Hz high-pass filter - Spectrally filtered EEG data were re-referenced to common average - Bad channels were rejected based on thresholding spatial correlations among channels - Subjects with more than 20% bad channels were discarded - Rejected channels were interpolated from EEG of adjacent channels via spherical spline interpolation - Remaining artifacts were removed using ICA - EEG data re-referenced to common average	SELSER Channel-level alpha band power Theta Coherence Band power features of latent signals extracted with ICA or PCA	*Alpha band power and theta coherence as described above* *SELSER* - spatial filter transforms multi-channel EEG data into a single latent signal, where the power is used as a feature - model fitting is done under a sparse constraint on the number of spatial filters, which reduces dimensionality *Latent signals extracted with ICA or PCA* - eigenvalues of the covariance matrix to reduce dimensionality	SELSER	Best performance using SELSER on alpha frequency range eyes-open rsEEG data *(feature importance was not reported)*
Zhdanov [41]	0.05–100 Hz bandpass filter Filtering performed using 2nd order Butterworth filters applied to the data in forward and reverse direction, to eliminate phase distortion Data pre-processed with EEGLAB toolbox Channels contaminated by large sporadic artifact were identified by human analyst and deleted EEG data bandpass filtered 1–80 Hz Notch-filtered at 60 Hz	Electrode-level spectral features Source-level spectral features Multiscale-entropy-based features Microstate-based features	*Electrode-level spectral features* - EEGLAB function *spectopo* to obtain power spectrum - log-transformed absolute power obtained for each channel - For each pair, absolute power at left electrode divided by right, resulting in 25 features for each band *Source-level spectral features* eLORETA algorithm as implemented by LORETA-KEY software Following regions selected on basis of prior literature: ACC, rACC, and mOFC *Multiscale-entropy-based features* - Quantifies variability of time series by estimating predictability of amplitude patterns across a time series - Two consecutive data points were used for data matching, and points were considered to match if their absolute amplitude difference was <15% of the standard deviation of the time series. *Microstate-based features* - Implemented using CARTOOL - *average duration*: average amount of time a microstate class remains stable when it appears (in ms) - *frequency*: occurrence of each microstate class per second - *coverage*: % of recording covered by each microstate class	Unpaired 2-tailed *t* test	MSE asymmetry features—C3/C4 (baseline) MSE asymmetry features—FC3/FC4 (baseline) MSE asymmetry features—T7/T8 (week 2) MSE asymmetry features—CP3/CP4 (week 2) Electrode-level spectral asymmetry—P3/P4 alpha low (baseline) Electrode-level spectral asymmetry —T7/TP8 theta (week 2) Electrode-level spectral asymmetry —F7/F8 beta mid (week 2) Source-level spectral features—alpha high ACC, rACC (week 2)

*ACC* anterior cingulate cortex, *rACC* rostral anterior cingulate cortex, *ANN* artificial neural network, *CCA* canonical correlation analysis, *Coh* coherence, *eLORETA* exact low-resolution brain electromagnetic tomography, *FDR* Fisher’s discriminant ratio, *FIR* finite impulse response, *FFT* fast Fourier transformation, *GMM* Gaussian mixture model, *ICA* independent component analysis, *KFD* Katz fractal dimension, *LASSO* least absolute shrinkage and selection operator, *LCMV* linearly constrained minimum variance, *LightGBM* light gradient boosting machine, *LZC* Lempel–Ziv complexity, *MARA* multiple artifact rejection algorithm, *MNI* Montreal Neurological Institute, *mOFC* medial orbitofrontal cortex, *MRF* middle right frontal, *mRMR* maximum relevance minimum redundancy, *MSC* magnitude squared coherence, *PCA* principal component analysis, *PSD* power spectral density, *rACC* rostral Anterior Cingulate Cortex, *rsEEG* resting-state EEG, *SELSER* sparse EEG latent space regression.

Corlier and colleagues predicted treatment response to open-label 10 Hz rTMS applied to the left dorsolateral prefrontal cortex (DLPFC) in a sample of 109 patients with MDD. Treatment response was defined as a decrease of ≥40% in post-treatment 30-item inventory of depressive symptomatology—self-rated (IDS-30) scores. Extracted features comprised changes in neurophysiological connectivity in the individual alpha frequency (IAF) band in response to rTMS stimulation. Using an elastic net model, which provides an embedded form of feature selection, the authors reported an accuracy of 61.8–69.3%, with the best performance using alpha spectral coherence features, defined as spectral correlation in the alpha frequency band. Of note, the same model showed 77% accuracy in a unilateral treatment subgroup [28].

Furthermore, Erguzel and colleagues developed a model to predict antidepressant response to 20 sessions of adjunctive 25 Hz rTMS applied to the left PFC in a sample of 147 individuals with MDD. Responder status was operationalized as a ≥50% reduction in Hamilton Depression Rating Scale (HAM-D) scores at the end of treatment. The best performance was observed in a Support Vector Machine (SVM) model in the theta frequency band across prefrontal regions using cordance features, which combines absolute and relative resting EEG activity, with an accuracy of 86.4% [32]. Additionally, Hasanzadeh et al. developed a model to predict response to 5-sessions of 10 Hz rTMS applied to the left DLPFC among 46 patients with MDD. Treatment response was defined as ≥50% decrease in BDI-II or HAMD-24 scores or by BDI ≤ 8 (HAMD-24 ≤ 9) which indicates remission. Using a *k*-Nearest Neighbors (*k*-NN) model, the best performance was observed using Lempel–Ziv complexity features in the beta frequency band, which counts the number of distinct segments in the signal, with an accuracy of 82.6%. [32].

Another study [28] predicted treatment response (≥50% improvement in HAMD-17) in an 18-session open-label trial of 25 Hz rTMS to the left prefrontal cortex, comprising 55 patients with MDD using cordance features in the delta and theta frequency bands, resulting in 89.09% accuracy. However, since accuracy was assessed using internal *k*-fold cross-validation alone, performance may be over-optimistic. In another study, treatment response was predicted within a 15-session open-label trial of 10 Hz left prefrontal rTMS in 39 patients with MDD using theta, upper alpha, and upper gamma power and connectivity, as well as theta-gamma coupling features, resulting in an accuracy of 91% [25]. Similarly, in another study using the same experimental design in 32 patients with MDD, treatment response was predicted using theta and alpha power and connectivity, frontal theta cordance, and alpha peak frequency, resulting in an accuracy of 86.66% [26]. Furthermore, other studies with insufficient sample sizes predicted response to tDCS [41], and rTMS [42], as further described in Supplementary Table S1.

Across neurostimulation trials, important features included absolute and relative power in frontal electrodes (alpha and theta band), connectivity measures (theta and gamma), spectral entropy, and cordance features across alpha, theta, delta, and gamma frequency bands. As described elsewhere [43], spectral entropy of a signal is a measure of its spectral power distribution and is based on Shannon’s entropy. With respect to important channels, one study [28] found Fp1, Fp2, F3, F7, and F8 in the theta frequency band to be important features following feature selection, and these same features were used in a follow-up study [30] by the same group, largely maintaining model accuracy (89.12% vs. 78.3–86.4%, respectively). One study [32] compared nonlinear, power spectral density, bi-spectral features, and cordance, with the best performance observed when restricting features to power over all 19-channels in delta, theta, alpha, and beta frequency ranges [33]. Furthermore, another study [26] found enhanced theta power at Fz to differ significantly between responders and non-responders (*F*1 = 8.577, *p* = 0.006), however, no main effect for frontal-midline theta power was observed in a follow-up study [27]. Furthermore, three studies [26, 27, 33] did not report feature selection methods, and surprisingly, no studies compared multiple feature selection methods. Further details can be observed in Table 2.

### Studies predicting clinical response to pharmacological treatment

Seven studies developed predictive models of clinical response to pharmacological treatment [34–41]. Among these, three studies assessed treatment response to various classes of antidepressants within randomized double-blind trials [35–38], one assessed response within a randomized trial of ketamine or placebo [34], one assessed response in an open-label trial of an SSRI [38], and two other studies assessed response to sertraline [40], and escitalopram [41], respectively.

Wu and colleagues developed a machine learning model known as Sparse EEG Latent SpacE Regression (SELSER), applied to alpha, beta, delta, and gamma frequency bands, to predict antidepressant treatment response using resting-state EEG. SELSER was first trained on data from the largest neuroimaging-couped placebo-controlled randomized clinical study of antidepressant efficacy, comprising 309 patients. The generalizability of the antidepressant signature was tested in two independent samples of depressed patients treated with antidepressants, and another sample of patients treated with rTMS to assess the specificity of SELSER’s signature for predicting response to antidepressants. Response was defined according to HAMD-17 change scores at the end of treatment. SELSER was shown to generalize across antidepressant datasets, with an *R*^2^ of 0.60 in predicting response to sertraline, and an *R*^2^ of 0.41 in predicting response to placebo, respectively [40].

Cao and colleagues developed a machine learning model to predict rapid antidepressant response to ketamine in a sample of 55 patients with treatment-resistant depression. Response was defined as ≥45% reduction in depressive symptoms (HAMD-17) 240 min following infusion. Using EEG power in delta, theta, lower-alpha, and upper alpha bands, as well as alpha asymmetry in frontal electrodes as candidate features, the best performance was observed using SVM with a radial kernel, resulting in an accuracy of 78.4% [34].

De la Salle and colleagues developed a model to predict response within a double-blinded 12-week trial of escitalopram, bupropion, or combined treatments, in 47 patients with treatment-resistant depression. Clinical response was defined as a ≥50% reduction in MADRS scores from baseline, and remitters were operationalized as those with ≤10 MADRS scores at posttreatment. Within a logistic regression model, change scores in middle right frontal cordance and prefrontal cordance across delta, theta, alpha, and beta frequency bands resulted in an accuracy of 74% and 81% in predicting clinical response, respectively. Similarly, clinical remission could be predicted with 70% accuracy using prefrontal cordance, however, middle right frontal cordance features were not discriminative (51% accuracy). It is important to note that EEG features alone resulted in better accuracy (74–81%) than clinical features alone (66%) or a combined model of EEG and clinical features (64–66%) [36].

Furthermore, Zhdanov et al. predicted antidepressant response to an 8-week open-label trial of escitalopram (10–20 mg) in a sample of 122 patients with MDD. Patients were classified as responders if they showed ≥50% reduction in Montgomery-Asberg Depression Rating Scale (MADRS) scores at the end of treatment. Of note, four classes of features were used, comprising electrode-level and source-level spectral features, multiscale-entropy-based features, and microstate-based features, as described in further detail within Supplementary Table 1. Using baseline EEG features alone, their SVM model showed an accuracy of 79.2%. Performance improved slightly when adding EEG features from the second week of treatment, with an accuracy of 82.4% [41].

In another study, Rajpurkar and colleagues predicted improvement in individual symptoms within the HAM-D from baseline to week 8 within a randomized trial of escitalopram, sertraline, or extended-release venlafaxine in a sample of 518 patients with MDD. Pre-treatment EEG candidate features included frontal alpha asymmetry, occipital beta asymmetry, and the ratio of beta/alpha and theta/alpha band power for each electrode. Using a gradient boosting machine (GBM) model with embedded feature selection, the authors reported an *R*^2^ of 0.375–0.551, with the best performance using EEG and baseline symptom features [39]. Other studies predicted response to various classes of antidepressants, resulting in an accuracy of 88% [37], treatment remission, resulting in an accuracy of 64.4% [35], and treatment response to an open-label trial of an SSRI, resulting in an accuracy of 87.5% [38].

Across medication trials, important features included alpha, theta, and gamma power in frontal electrodes, coherence between frontal and temporal electrodes, change scores in delta power, the ratio of alpha and theta power in temporal electrodes, and asymmetry between hemispheres. With respect to important channels, two studies [34, 39] found Fp2 absolute theta to be among the top ten features to predict response to SSRIs/SNRI, and ketamine, respectively. Additionally, two studies [37, 39] showed baseline power at F7 to be an important feature, although in different frequency bands, corresponding to alpha, beta, and gamma, respectively. Overall, studies varied widely in the number of electrodes, electrodes of interest, and feature extraction methods, which preclude a set of well-elucidated individual biomarkers of treatment response.

### Improvements in model accuracy by incorporating EEG features

Additionally, we sought to investigate the contribution of EEG-based features to predictive accuracy in cases where clinical variables were also incorporated into predictive models of treatment response. However, only six studies [26–28, 37, 39, 41] (40%) used both EEG and clinical candidate features within model development. Among them, only one [28] reported differences in model accuracy between EEG features, clinical features, and combined models. Corlier and colleagues reported that alpha spectral correlation features predicted treatment response with 69.3% accuracy (Sensitivity: 67.1%, Specificity: 70.9%), while baseline IDS-30 scores predicted treatment response with 75.1% accuracy (Sensitivity: 64.1%, Specificity: 83.6%). Combining both features lead to greater model performance, with an accuracy of 79.2% (Sensitivity: 75.7%, Specificity: 81.9%) [28].

### Quality metrics

Overall, samples used to develop models were small, with a median sample size of 55 among studies predicting response to neurostimulation, and 86.5 among studies predicting response to antidepressant medication, respectively. Quality metrics were assessed using the QUADAS-2 [24], and a quality assessment instrument specific to machine learning. These quality assessment metrics can be found in Supplementary Table 2, and the Supplementary Material, respectively. The QUADAS-2, as described elsewhere [24], evaluates the risk of bias according to the domains of patient selection, index test, reference standard, and flow and timing. Overall, most studies showed a low risk of bias according to patient selection, how treatment response was defined, and the time interval between EEG assessments and treatment follow-up. However, 7 of 15 (46.6%) [26, 27, 29, 30, 32, 33, 38] showed a high risk of bias in reference standards for model development, which included a lack of training/testing sets, and a lack of blinded assessment to treatment allocation when collecting symptom scales and EEG data.

With respect to the machine learning quality assessment, the median score for neurostimulation studies was 5/9 (55.5%), and the median score for psychiatric medication studies was 6.5/9 (72.2%), respectively. Only two studies [27, 34] discussed methods to address the class imbalance, which occurs in classification models where there is a disproportionate ratio of observations in each class (e.g., responders vs. non-responders). Moreover, several studies [26, 27, 29, 30, 32, 33, 35–37, 39] evaluated performance using cross-validation in the absence of training and testing sets, which increases the risk of model overfitting and may lead to biased results.

### Meta-analyses of predictive models of treatment response using EEG

Within the fifteen studies included in the systematic review, seven predicted treatment responses to rTMS [26–30, 32, 33], and eight predicted responses to antidepressant treatments (ketamine, escitalopram, sertraline, escitalopram, bupropion, and venlafaxine), respectively [34–41]. Among them, 12 involved binary classification models [26–29, 32–38, 41] (response vs. non-response) and reported summary statistics required to pool predictive accuracy. A detailed summary of performance metrics across models can be found in Supplementary Table S4. The accuracy of treatment response prediction models in MDD across 758 patients was pooled in a random-effects model using an inverse variance method with a restricted maximum likelihood estimator to calculate the heterogeneity variance *τ*^2^. Furthermore, Knapp–Hartung adjustments were used to calculate the confidence interval around the pooled effect.

**Fig. 1:**
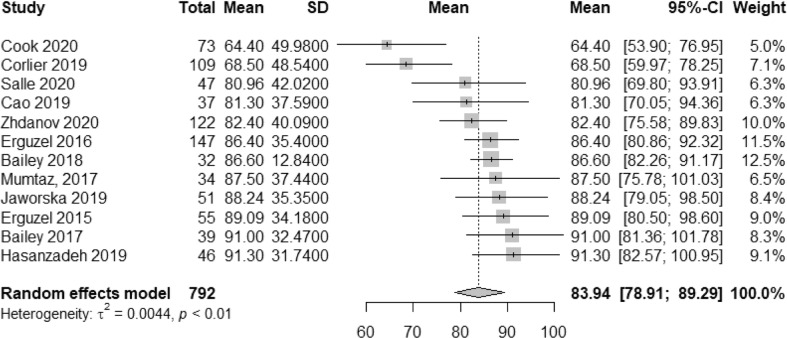
Pooled effects of treatment response accuracy using EEG. Pooled accuracy of treatment response prediction models in Major Depressive Disorder across 792 patients within a random-effects model using a restricted maximum-likelihood estimator to calculate the heterogeneity variance *τ*^2^. Model accuracy across studies was used, in conjunction with standard deviation, calculated by multiplying the standard error by the square root of the sample size (SD = SE × √*n*). Knapp–Hartung adjustments were used to calculate the confidence interval around the pooled effect. The average accuracy across models was 83.94% (95% CI: 78.91–89.29), with a heterogeneity variance *τ*^2^ of 0.0044.

**Fig. 2:**
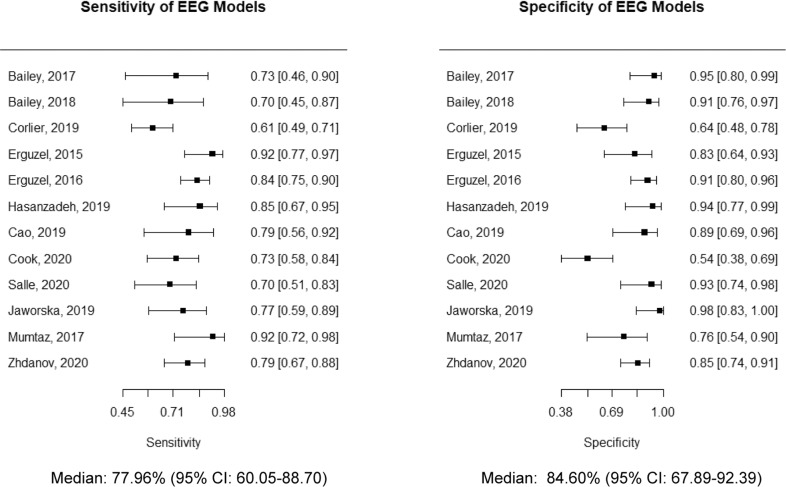
Sensitivity and specificity across models. A calculation of the sensitivity and specificity summary statistics across 12 studies using the frequencies of true positives, false negatives, false positives, and true negatives, using the *madad* function in the mada package in R. Overall, the balanced accuracy (sensitivity + specificity/2) across studies was 81.28%. Across studies, model sensitivity was lower than specificity, suggesting that predictive models of treatment response using EEG overall show better performance in identifying true non-responders to treatment (specificity), relative to true responders to treatment (sensitivity).

Overall, across six studies comprising 438 patients with MDD, the pooled accuracy of treatment response prediction using EEG was 83.93% (95% CI: 78.90–89.29), with a heterogeneity variance *τ*^2^ of 0.0044 (95% CI: 0.0009–0.0296), as depicted in Fig. 1. Moreover, the median sensitivity across studies was 77.96% (95% CI: 60.05–88.70), and median specificity was 84.60% (95% CI: 67.89–92.39), respectively, as shown in Fig. 2. Additionally, as shown in Table 3, the AUC was 0.850 (95% CI: 0.747–0.890), with a pAUC of 0.777, whereas the total DOR was 23.49 (95% CI: 10.40–52.02), with a posLR of 5.232 (95% CI: 3.15–8.67), and negLR of 0.271 (95% CI: 0.195–0.376), respectively. Briefly, DOR is a ratio of the odds of testing positive (e.g., predicted as a responder) when reaching therapeutic response to treatment, relative to the odds of testing positive (e.g., predicted as a responder), when failing to respond to treatment, although this metric is also dependent on prevalence [42]. Further information regarding this metric can be found elsewhere [43]. Similarly, posLR describes the probability of testing positive divided by the probability a positive test would be expected in a negative case, whereas negLR is defined as the opposite. A posLR of 10 or more and a negLR of 0.1 or less are generally deemed to be informative tests. Additionally, considering potential study heterogeneity across treatment modalities, a subgroup analysis was performed for rTMS and antidepressant models, where these outcomes were assessed separately, as shown in Supplementary Figs. S1–S4.

**Table 3:** Model performance metrics across EEG models.

(a)						
Authors	Sensitivity	2.5%	97.5%	Specificity	2.5%	97.5%
Bailey [26]	0.731	0.460	0.896	0.946	0.798	0.988
Bailey [26]	0.700	0.448	0.870	0.914	0.758	0.973
Corlier [28]	0.607	0.494	0.709	0.643	0.477	0.780
Erguzel [29]	0.919	0.772	0.975	0.827	0.643	0.927
Erguzel [31]	0.841	0.665	0.945	0.938	0.769	0.985
Hasanzadeh [33]	0.854	0.665	0.945	0.938	0.769	0.985
Cao [34]	0.794	0.558	0.922	0.886	0.694	0.964
Cook [35]	0.731	0.576	0.845	0.542	0.383	0.692
Salle [36]	0.696	0.511	0.834	0.929	0.741	0.983
Jaworska [37]	0.768	0.585	0.886	0.980	0.834	0.998
Mumtaz [38]	0.921	0.719	0.982	0.763	0.539	0.899
Zhdanov [41]	0.791	0.666	0.878	0.846	0.742	0.913
Average	0.776	0.600	0.892	0.846	0.678	0.923
Test for equality of sensitivities: *X*^2^ = 23.09, *p*-value = 0.017						
Test for equality of specificities: *X*^2^ = 46.23, *p*-value = 0.00000294						
Correlation of sensitivities and false-positive rates: Rho = −0.203 (−0696 to 0.420)						
Total DOR: 23.49 (95% CI: 10.40–52.02), *τ*^2^ = 1.395 (95% CI: 0.00–2.13)						
posLR: 5.232 (95% CI: 3.15–8.67), *τ*^2^ = 0.502 (0.00–1.24)						
negLR: 0.271 (95% CI: 0.195–0.376), *τ*^2^ = 0.190 (0.00–0.495)						
AUC: 0.850 (95% CI: 0.747–0.890); pAUC: 0.777						

(b)			
Authors	Mean accuracy	95% CI	%W (random)
Bailey [26]	91.0	81.34–100	8.3
Bailey [26]	86.6	82.23–91.16	12.5
Corlier [28]	68.5	59.96–78.24	7.1
Erguzel [29]	89.0	80.49–98.59	9.0
Erguzel [31]	86.4	80.86–92.31	11.5
Hasanzadeh [33]	91.3	82.57–100	9.1
Cao [34]	81.3	70.04–94.36	6.3
Cook [35]	64.4	53.89–76.94	5.0
Salle [36]	80.9	69.79–93.91	6.3
Jaworska [37]	88.2	79.05–98.49	8.4
Mumtaz [38]	87.50	75.77–100	6.5
Zhdanov [41]	82.4	75.58–89.83	10.0
Random effects model			
Mean = 83.93% (95% CI: 78.90–89.29)			

A summary of performance metrics across all predictive models of treatment response using EEG.

(a) The madad function in the “mada” package was used to calculate the sensitivity, specificity, and partial Area-Under-The-Curve (AUC) across studies, while the maduani function was used to calculate the Diagnostic Odds Ratio (DOR), positive likelihood ratio (posLR), and negative likelihood ratio (negLR). AUC was calculated using the AUC_boot function in dmetatools, with an alpha of 0.95 and 2000 bootstrap iterations. Overall, the balanced accuracy (sensitivity + specificity/2) was 81.1%.

(b) The metamean function in the “meta” package was used to pool accuracy across studies in a random effects model using an inverse variance method with Knapp–Hartung adjustments to calculate the confidence interval around the pooled effect. Across models, overall model accuracy was 83.93% (95% CI: 78.90–89.29).

### Efficacy of predicting treatment response to rTMS

Across six studies [26–29, 32, 33], comprising 438 patients, the pooled accuracy of rTMS treatment response prediction using EEG was 85.70% (95% CI: 77.45–94.83), with a heterogeneity variance *τ*^2^ of 0.0051 (95% CI: 0.0004: 0.0668). The median sensitivity across studies was 79.4% (95% CI: 58.65–90.80) and median specificity was 92.05% (95% CI: 81.70–99.30), respectively. Overall, the AUC across studies was 0.895 (95% CI: 76.07–93.99), with a partial AUC of 0.821, a DOR of 35.48 (95% CI: 7.805–161.364, *τ*^2^ = 2.797), posLR of 7.098 (95% CI: 2.843–17.725, *τ*^2^ = 0.915), and negLR of 0.234 (95% CI: 0.122–0.448, *τ*^2^ = 0.478), respectively.

A test for equality of proportions with a continuity correction of 0.5 yielded a Chi-squared (*X*^2^) value of 20.05 (*p* = 0.0012) and 20.62 (*p* = 0.00095) for sensitivities and specificity, respectively. Moreover, a moderate negative correlation was observed between sensitivities and false-positive rates (Rho = −0.526 (95% CI: −0.937 to 0.498). Further details can be observed in Supplementary Figs. S1 and S3.

### Efficacy of predicting treatment response to antidepressants

Across five studies [35–38, 41], comprising 325 patients, the pooled accuracy of antidepressant treatment response prediction using EEG was 81.41% (95% CI: 71.09–92.23), with a heterogeneity variance *τ*^2^ of 0.0052 (95% CI: 0.00–0.11), as depicted in Supplementary Fig. S2. The median sensitivity across studies was 77.78% (95% CI: 61.14–88.50), and median specificity was 82.06% (95% CI: 65.54–95.24), respectively. Overall, the AUC of studies predicting response to antidepressant medications was 0.764 (95% CI: 0.710–0.899) with a partial AUC of 0.756. Furthermore, the overall DOR was 19.02 (95% CI: 5.51–65.61), with a posLR of 4.30 (95% CI: 1.92–9.64), and negLR of 0.296 (95% CI: 0.208–0.422). A test for equality of proportions with a continuity correction of 0.5 yielded an *X*^2^ of 3.8 (*p* = 0.434) for sensitivities and an *X*^2^ of 23.67 (*p* = 0.0000927) for specificities, respectively. Moreover, a weak negative correlation of sensitivities and false-positive rates was observed across studies (Rho = −0.016, 95% CI: −0.886 to 0.879). Further details can be observed in Supplementary Figs. S2 and S4.

Considering the small number of antidepressant studies, we performed another meta-analysis with the addition of three studies [44–46] that were excluded due to small sample size (*N* ≤ 30), increasing the total sample to 402 patients with MDD. This resulted in a pooled accuracy of 84.52% (95% CI: 77.67–91.98, *τ*^2^ = 0.0034), median sensitivity of 82.07% (95% CI: 60.96–91.72), median specificity of 84.47% (95% CI: 65.28–92.55), and AUC of 0.794 (95% CI: 0.728–0.887). Additionally, the DOR was 28.98 (95% CI: 9.95–84.4), with a posLR of 5.20 (95% CI: 2.67–10.15), and negLR of 0.26 (95% CI: 0.19–0.37). Further details can be found in Supplementary Fig. S5.

## Discussion

While there is a great deal of promise in using EEG within machine learning models to predict treatment response in MDD, there does not appear to be a consensus on collection methods, consistent physiological markers of response to antidepressants, or rTMS across studies. Given the complexity of MDD, and the likelihood of heterogeneity in important features across patients, the field may require a conceptual shift away from the search for singular biomarkers, towards the use of composite features, identified using multivariate models. As such, it may be the case that no singular neurophysiological biomarker will demonstrate the sensitivity and specificity required to guide treatment selection in MDD. Rather, a composite biomarker comprising a series of distinct, but mutually informative features, may serve to both improve our mechanistic understanding of treatment response, and appropriately model this phenomenon. However, it is important to highlight that multimodal feature combinations carry several additional considerations. Namely, if complex approaches such as source localization are required to provide meaningful accuracy, this may provide a significant challenge in the clinical implementation of such models. Additionally, while resting-state features provide greater scalability relative to EEG activation patterns during specific tasks, the latter may inform features that could perhaps be more sensitive and specific in modeling clinical improvement in response to a given treatment.

### Model performance across meta-analyses

Overall, model performance in predicting response to rTMS (accuracy = 85.70%, 95% CI: 77.45–94.83; AUC = 0.895, 95% CI: 76.07–93.99, DOR = 35.48, 95% CI: 7.805–161.364) was greater than predicting response to antidepressants (accuracy = 81.41%, 95% CI: 71.09–92.23; AUC = 0.764, 95% CI: 0.710–0.899, DOR = 19.02, 95% CI: 5.51–65.61), even after the addition of three excluded studies to increase the sample size (accuracy = 84.52%, 95% CI: 77.67–91.98; AUC = 0.794, 95% CI: 0.776–0.919; DOR = 28.98, 95% CI: 9.95–84.4). This was also found relative to a total model including 12 studies (*N* = 792) across all rTMS and medication trials (accuracy = 83.93%, 95% CI: 78.90–89.29; AUC: 0.850, 95% CI: 0.600–0.887; DOR = 23.49, 95% CI: 10.40–52.02).

There are several potential contributing factors to this finding, as models that predicted response to rTMS utilised data from open-label trials that lacked an adequate sham condition. However, it is posited that this may be reflective of very specific targets across rTMS studies, since all involved high-frequency stimulation (10–25 Hz) to the DLPFC. Moreover, it is speculated that EEG, which measures electrical activity primarily near the surface of the cortex, is assessing neural networks that are more likely to be directly targeted by rTMS. Conversely, with respect to pharmacotherapy, the effect is much more indirect and potentially dependent on other factors that EEG cannot access such as hepatic metabolism, and pharmacokinetic interactions.

Interestingly, across all four meta-analyses, model specificity (82.06–92.05%) was notably greater than model sensitivity (77.96–82.07%), even when considering the upper and lower bounds of the confidence intervals. This suggests that across all treatment modalities, including rTMS, antidepressants, and a combined model, EEG features are better able to capture predictors of clinical non-response to treatment, rather than predictors of clinical response. As such, it is possible that EEG may show greater utility in determining whether a patient will not respond to a given intervention at baseline. However, prospective validation with large samples in independent cohorts will be necessary to determine the reliability of this finding.

Additionally, the rTMS model showed a higher DOR (DOR = 35.48, 95% CI: 7.805–161.364; *τ*^2^ = 2.797, 95% CI: 0.00–8.402), relative to the total model (DOR = 23.49, 95% CI: 10.40–53.02; *τ*^2^ = 1.395, 95% CI: 0.00–2.13), and antidepressant model (DOR = 19.02, 95% CI: 5.51–65.61); *τ*^2^ = 1.27, 95% CI: 0.00–14.79), respectively. This indicates that the odds for positivity among individuals who respond to treatment are 35 times higher than the odds for positivity among individuals who will not respond to treatment. However, it is important to highlight that a large upper and lower bound of the confidence interval was observed across rTMS studies, as well as greater heterogeneity.

### Independent validation, feature replicability, and clinical outcomes

Nonetheless, there is a need for greater emphasis on testing model performance with independent samples, greater consistency in sample collection and model development, and an increased focus on replicating features identified in previous models. Additionally, nine studies [26–30, 32, 33, 36, 38] (60%) included in the present meta-analysis and systematic review did not test accuracy in holdout data, relying instead on internal cross-validation, which may lead to overoptimistic performance metrics. Furthermore, most studies (57.1%) utilised data from open-label trials lacking adequate double-blind procedures, and as such, there is a risk of bias pertaining to the scoring and interpretation of treatment response. There also remains an unmet need for prospective studies that compare features between models of treatment response and remission outcomes. Thus far, only one study [36] has assessed both outcomes, although it did not report a difference in top features between these models. It remains to be determined whether there are reproducible features that are specific to reaching the threshold for treatment response, relative to treatment remission.

### Definitions of clinical response

Most studies contained in the present review (86.6%) used binary classification models to discriminate treatment responders’ treatment from non-responders. As detailed further in Supplementary Fig. S4, studies varied in terms of the specific clinical scale and change-score thresholds that constituted treatment response. Overall, four studies (26.6%) selected a ≥ 50% reduction in the HAMD-17 as the threshold of clinical response, while three studies (20%) defined clinical response as a ≥50% reduction in the MADRS. Large differences in treatment duration were also observed across trials. Importantly, greater standardization in how clinical response is defined is required to better assess the performance of prospective models, aid in the reproducibility of findings, and improve the likelihood of real-world clinical utility of ML models in psychiatry. Similarly, as described elsewhere [47], there is a lack of clear consensus on how treatment resistance is defined, which highlights the need for greater consistency across studies.

### Comparison of algorithms across studies

Furthermore, only three studies (20%) [32, 34, 37] assessed the performance of multiple algorithms, which limits a comparison of which algorithms tended to perform well. Considering this, two studies [48, 49] that were excluded due to insufficient sample size which assessed multiple algorithms were pooled with included studies to examine potential trends, comprising a total of five studies. Among them, SVM was compared alongside other algorithms such as random forest within five studies and resulted in the best performance in 60% of cases. In the other 40% of cases [37, 49], only composite accuracy across algorithms was reported. As described elsewhere [50], SVM is well suited to very high dimensional data, considering its use of support vectors, various available kernels, and computational efficiency in large datasets.

### Pre-processing strategies across studies

With respect to pre-processing strategies, all studies used a bandpass filter to limit included frequencies to a specific range, although studies varied widely (0.1–80 Hz) in terms of the upper and lower bounds. One study [41] also reported using a notch filter at 60 Hz, which attenuates frequencies in a specific range to very low levels. Furthermore, five studies [29, 30, 32, 37, 39] (33.3%) used independent component analysis to filter artifacts, and five [29, 30, 32, 34, 37] (33.3%) used a fast Fourier transform method. Other studies [33, 41] used available pre-processing packages, such as the EEGLAB toolbox available in the MATLAB programming language.

### Future perspectives

Prospectively, there is a need for models that examine the comparative effectiveness of multiple treatments across the same patients. Studies thus far have focused on predicting response to a specific intervention rather than treatment selection, and few have been replicated to see if a classification tool has worked in external independent datasets.

Furthermore, to facilitate EEG biomarkers of response to specific treatments, future studies may benefit from testing model performance on external datasets of other psychiatric medications or neurostimulation therapies. For example, Wu and colleagues assessed whether the algorithm SELSER, trained on SSRI datasets, could predict response to rTMS [40]. This approach may help highlight differences in important features to predict treatment response across psychiatric medications and provide an avenue to investigate potential neurophysiological mechanisms of action. Moreover, exploring whether models retain similar features and modest prediction accuracy when tested on external datasets of other interventions, may provide a way to identify generalizable EEG biomarkers that are related to therapeutic improvement or treatment resistance across disorders. Nonetheless, it may be more informative and realistic to focus on predictors of response to specific classes of medications and neurostimulation trials, to identify divergent mechanisms of therapeutic efficacy and treatment resistance. Either way, this will require careful consideration of differences in outcome instruments between datasets.

Surprisingly, in the present review, there was little overlap in top features between models, even when stratifying between rTMS or antidepressant trials. As such, there remains a critical need for a systematic comparison of several types of features in prospective models of treatment response and treatment selection to help guide prospective biomarker identification and validation. Of the 15 studies comprising the current review, only three [33, 34, 41] (20%) included three or more categories of candidate features during model development. For instance, Hasanzadeh and colleagues considered nonlinear, spectral entropy, and cordance features, and found that combining spectral entropy (beta and delta) and cordance features resulted in the highest performance [33]. Furthermore, Zhdanov and colleagues compared electrode-level spectral features, source-level spectral features, multiscale-entropy-based features, and micro-state-based features. Here, multiple-entropy-based features comprised the top 4 of 8 features in a model to predict response to 8-weeks of open-label escitalopram [41].

Apart from the categories of features used in the present review, as detailed in Table 2, prospective models may benefit from incorporating features derived from brain source localization methods. This process, as described elsewhere [51], involves predicting scalp potentials from current sources in the brain (forward problem) and estimating the location of the sources by measuring scalp potentials (inverse problem). These methods have the potential to improve the signal-to-noise ratio of extracted features and suppress volume conduction. However, they require an accurate head model which is often difficult to obtain. It remains unclear what the overall effectiveness of these methods is in the context of extracting meaningful features to predict treatment response.

Furthermore, as described in Supplementary Table S5, most predictive models have been developed using features derived from resting-state EEG. Only two studies [26, 38] (13.3%) have used task-specific EEG to derive features, which involved the Sternberg Working Memory Task and 3-Stimulus Visual Oddball Task. Apart from this, event-related potentials may prove useful, especially if we could identify stimuli that are sensitive to depressed and psychotic states. Moreover, none of the reviewed studies developed predictive models using a combination of resting-state and task-specific EEG. Incorporating both within the same model of treatment response may help inform potential mechanisms of action and yield more informative biomarkers. Additionally, no studies thus far have utilised intracranial EEG to predict treatment response in MDD. By placing electrodes directly on the surface of the brain, intracranial EEG provides a much cleaner signal, and by its nature, greater source localization [52]. While intracranial EEG is much more invasive relative to surface electrodes, it may be justified for severe cases of treatment resistance.

With respect to algorithm selection, SVM was found to perform well when comparisons against other algorithms were available. Apart from the approach of comparing performance across individual algorithms, stacked generalization [53] provides an alternative ensemble method to combine the predictions of two or more machine learning algorithms, while using another algorithm to learn how to combine their outputs. As described elsewhere [54], stacking can improve model performance over any single model contained in the ensemble. Additionally, stacking differs from the traditional bagging and boosting ensemble methods in that it typically uses different models that combine predictions from contributing models, rather than a series of decision trees, or models that comprise weak learners building upon the prediction of previous models, respectively. While two studies [37, 49] averaged results across models into a composite accuracy, to our knowledge, stacked generalization has not yet been explored in predictive models of treatment response using EEG.

Similarly, hyperparameter tuning, which involves selecting the optimal set of hyperparameters for a given model, remains an important consideration in model development [55]. While many software packages have default hyperparameter settings during cross-validation, searching the hyper-parameter space for the lowest loss-function, or best cross-validation score is recommended. Although an exhaustive search of the hyperparameter space is often computationally infeasible, there are several available methods such as a manual grid search, collaborative hyperparameter tuning [56], and Bayesian optimization [57].

As demonstrated in the current review, studies varied largely in the number of electrodes used, EEG systems, feature selection and extraction methods, and machine learning algorithms. Considering the heterogeneity observed across studies, large, standardized datasets must become available before this field can move ahead in a significant way. Importantly, there is a need for models developed using large well-characterized samples, with separate training, testing, and external validation datasets, to derive classification tools that can be useful clinically. Similarly, available repositories are needed to appropriately replicate models developed thus far, identify generalizable biomarkers of treatment response across interventions, and identify distinct neurophysiological markers that can help guide treatment selection in MDD.

## Supplementary information

Supplementary Figures

Supplementary Tables

Supplementary Material
