# Supplementary material for: Predicting treatment response using EEG in major depressive disorder: A machine-learning meta-analysis
Source: Transl Psychiatry. 2022 Aug 12;12:332. doi: 10.1038/s41398-022-02064-z (PMC9374666; doi:10.1038/s41398-022-02064-z)
Supplement: Supplementary file 1 — Supplementary Figures [file 41398_2022_2064_MOESM1_ESM.docx]

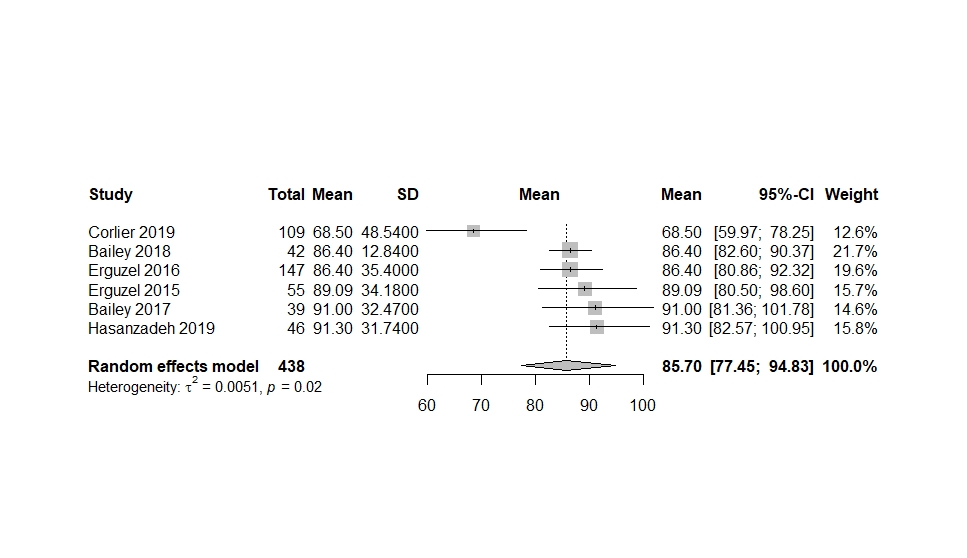


**Supplementary Figure S1: Pooled accuracy of rTMS treatment response prediction using EEG**

Pooled accuracy of rTMS treatment response prediction models in Major Depressive Disorder across 438 patients within a random-effects model using a restricted maximum likelihood estimator to calculate the heterogeneity variance τ^2^. Model accuracy across studies was used, in conjunction with standard deviation, calculated by multiplying the standard error by the square root of the sample size (SD = SE×√n). Knapp-Hartung adjustments were used to calculate the confidence interval around the pooled effect. The average accuracy across models was 85.70% (95% CI: 77.45-94.83), with a heterogeneity variance τ^2^ of 0.0051.


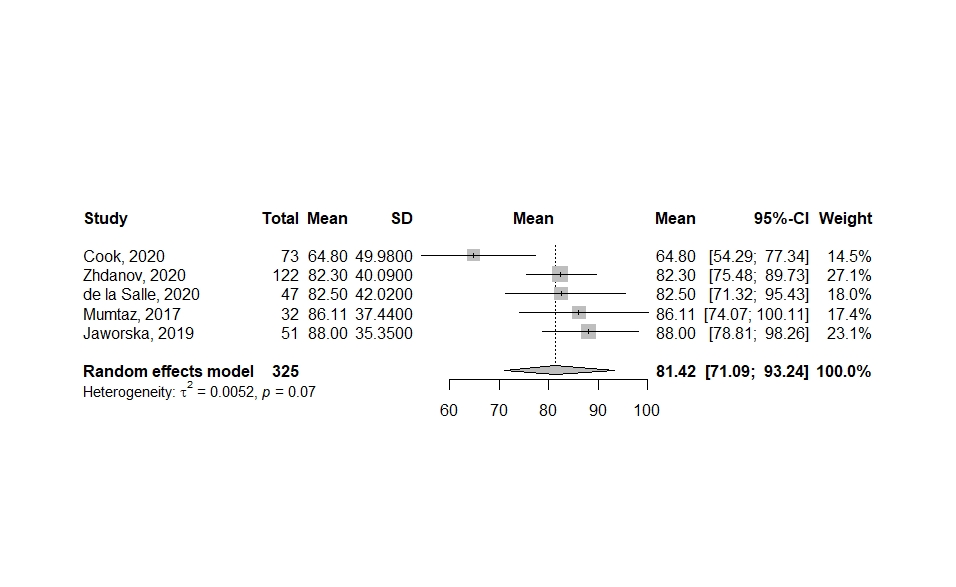


**Supplementary Figure S2: Pooled accuracy of antidepressant treatment response prediction using EEG**

Pooled accuracy of antidepressant treatment response prediction models in Major Depressive Disorder across 325 patients within a random-effects model using a restricted maximum likelihood estimator to calculate the heterogeneity variance τ^2^. Model accuracy across studies was used, in conjunction with standard deviation, calculated by multiplying the standard error by the square root of the sample size (SD = SE×√n). Knapp-Hartung adjustments were used to calculate the confidence interval around the pooled effect. The average accuracy across models was 81.42% (95% CI: 71.09-93.24), with a heterogeneity variance τ^2^ of 0.0052.


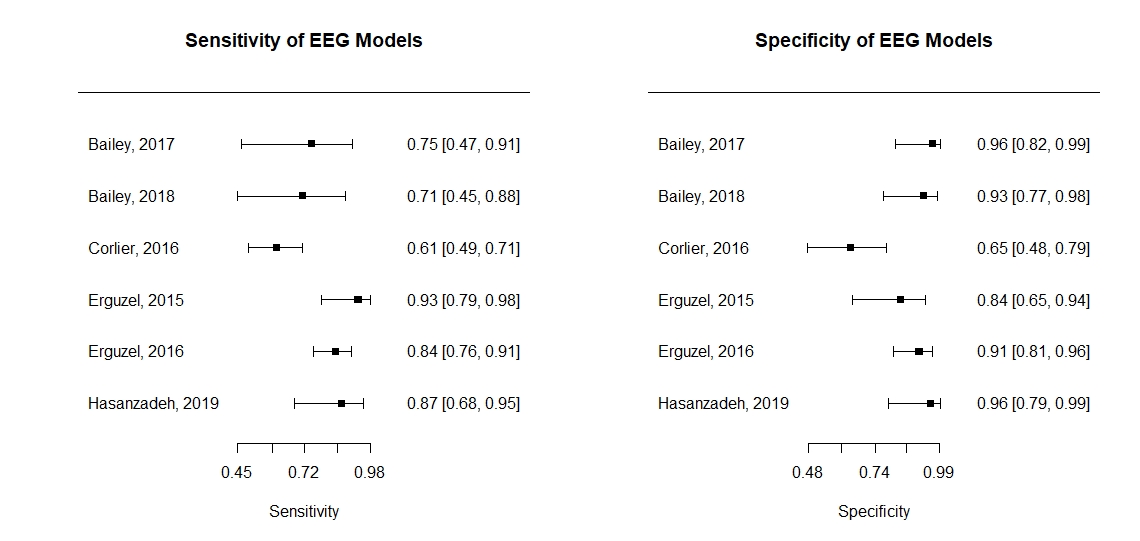


Median: 79.78% (95% CI: 58.65-90.8) Median: 92.05% (95% CI: 81.70-99.30)

**Supplementary Figure S3: Sensitivity and Specificity of rTMS Models**

A calculation of the sensitivity and specificity summary statistics across six studies using the frequencies of true positives, false negatives, false positives, and true negatives, using the *madad* function in the mada package in R. Overall, the balanced accuracy (sensitivity + specificity/2) across studies was 85.91%. Across studies, model sensitivity was lower than specificity, suggesting that predictive models of treatment response using EEG overall show better performance in identifying true non-responders to treatment (specificity), relative to true responders to treatment (sensitivity).


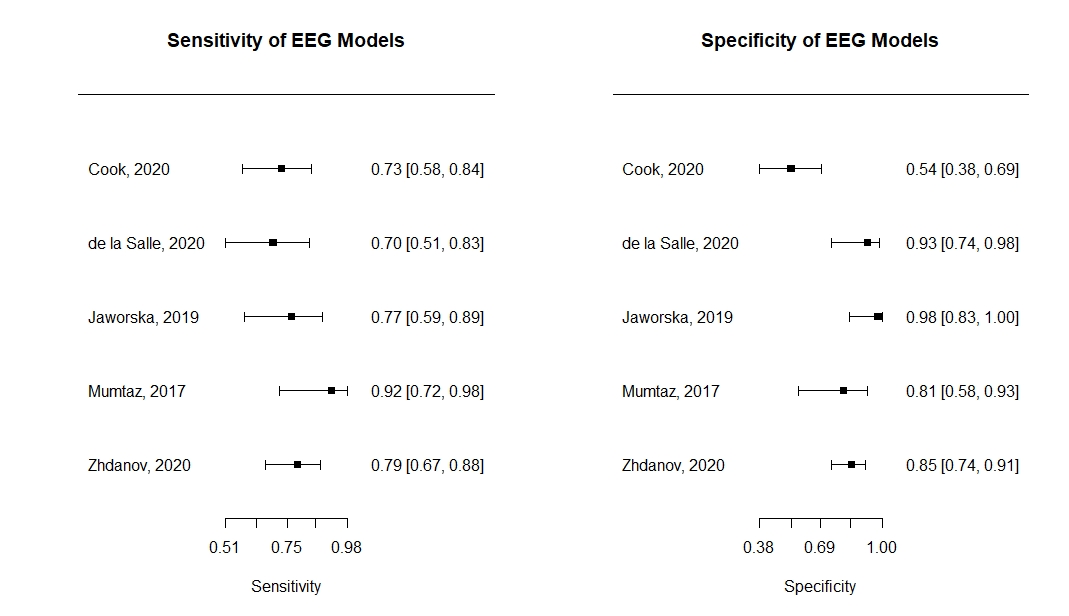


Median: 77.78% (95% CI: 61.14-88.50) Median: 82.06% (95% CI: 65.54-95.24)

**Supplementary Figure S4: Sensitivity and Specificity of antidepressant models**

A calculation of the sensitivity and specificity summary statistics across six studies using the frequencies of true positives, false negatives, false positives, and true negatives, using the *madad* function in the mada package in R. Overall, the balanced accuracy (sensitivity + specificity/2) across studies was 79.92%. Across studies, model sensitivity was lower than specificity, suggesting that predictive models of treatment response using EEG overall show better performance in identifying true non-responders to treatment (specificity), relative to true responders to treatment (sensitivity).


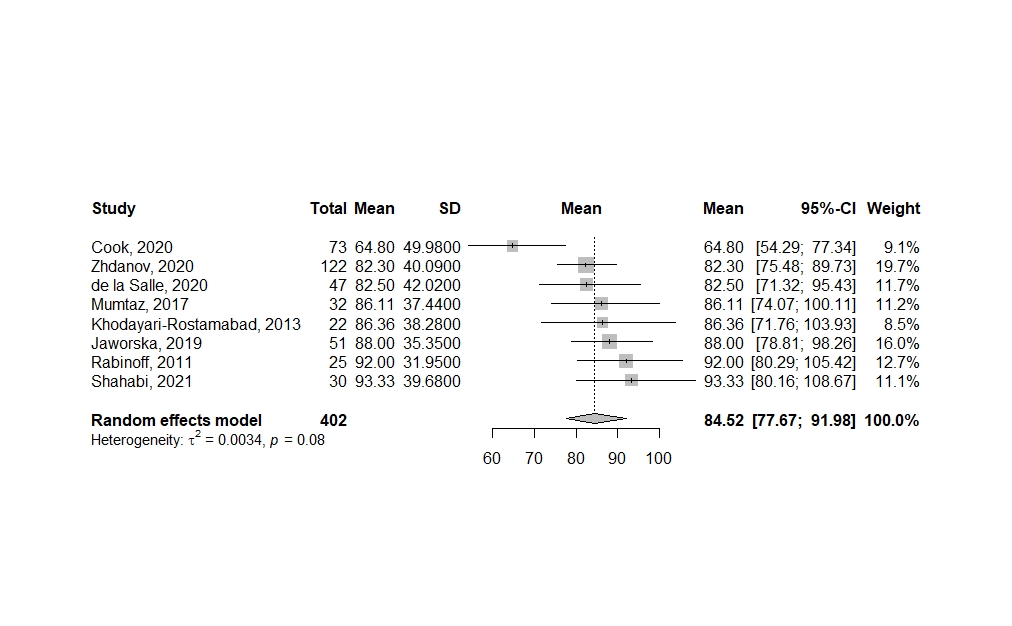


**Supplementary Figure S5:** Pooled accuracy of antidepressant models and excluded studies

Pooled accuracy of antidepressant treatment response prediction models in Major Depressive Disorder across 402 patients (325 patients from included studies, and 77 from excluded studies) within a random-effects model using a restricted maximum likelihood estimator to calculate the heterogeneity variance τ^2^. Model accuracy across studies was used, in conjunction with standard deviation, calculated by multiplying the standard error by the square root of the sample size (SD = SE×√n). Knapp-Hartung adjustments were used to calculate the confidence interval around the pooled effect. The average accuracy across models was 84.52% (95% CI: 77.67-91.98), with a heterogeneity variance τ^2^ of 0.0034.


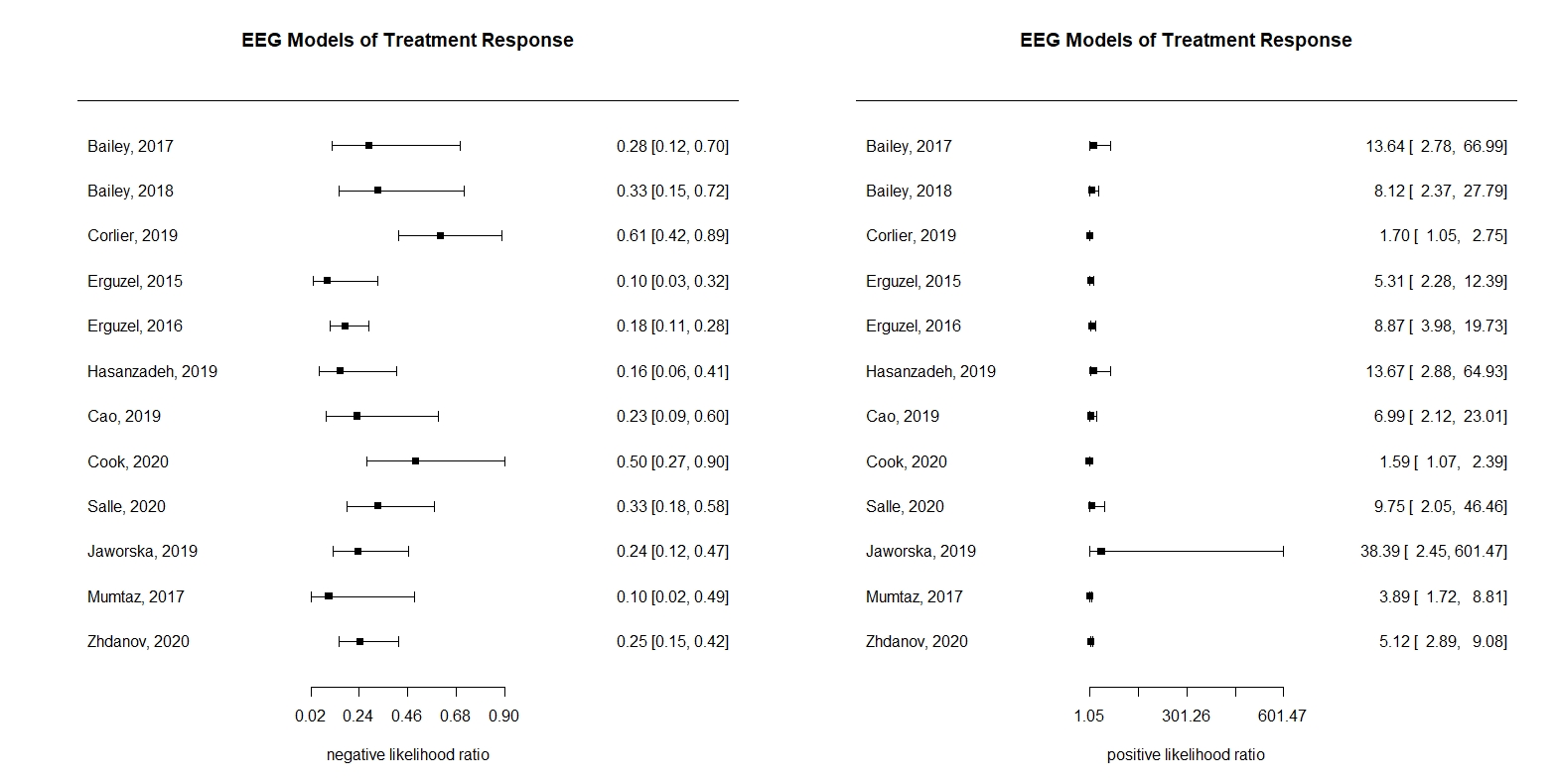


**Supplementary Figure S6: Negative and positive likelihood ratios of treatment response prediction using EEG**

A depiction of the negative and positive likelihood ratios across studies, alongside 95% confidence intervals. Overall, the diagnostic odds ratio across studies was 23.49 (95% CI: 10.40-52.02), with a positive likelihood ratio of 5.232 (95% CI: 3.15-8.67) and negative likelihood ratio of 0.271 (95% CI: 0.195-0.376).


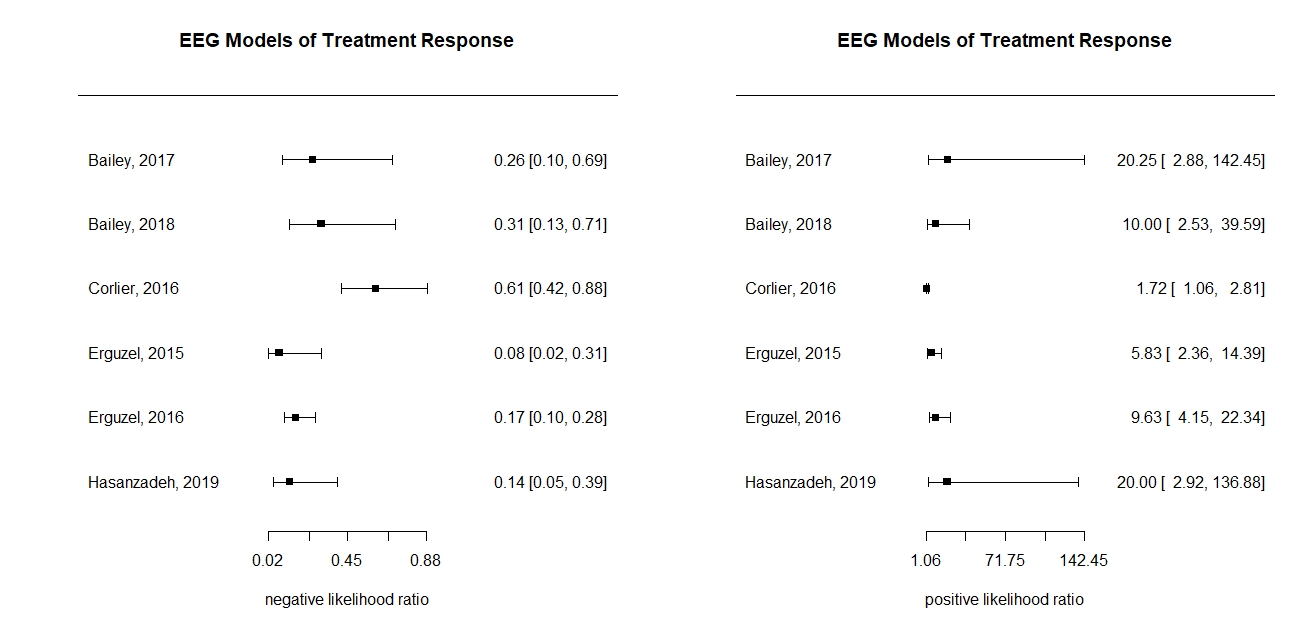


**Supplementary Figure S7: Negative and positive likelihood ratios of rTMS treatment response prediction using EEG**

A depiction of the negative and positive likelihood ratios across studies, alongside 95% confidence intervals. Overall, the diagnostic odds ratio (DOR) across studies was 35.48 (95% CI: 7.80-161.36), with a positive likelihood ratio of 7.098 (95% CI: 2.84-17.72) and negative likelihood ratio of 0.234 (95% CI: 0.122-0.448). Of note, a large upper and lower bounds of the 95% confidence interval was observed within the DOR and posLR.


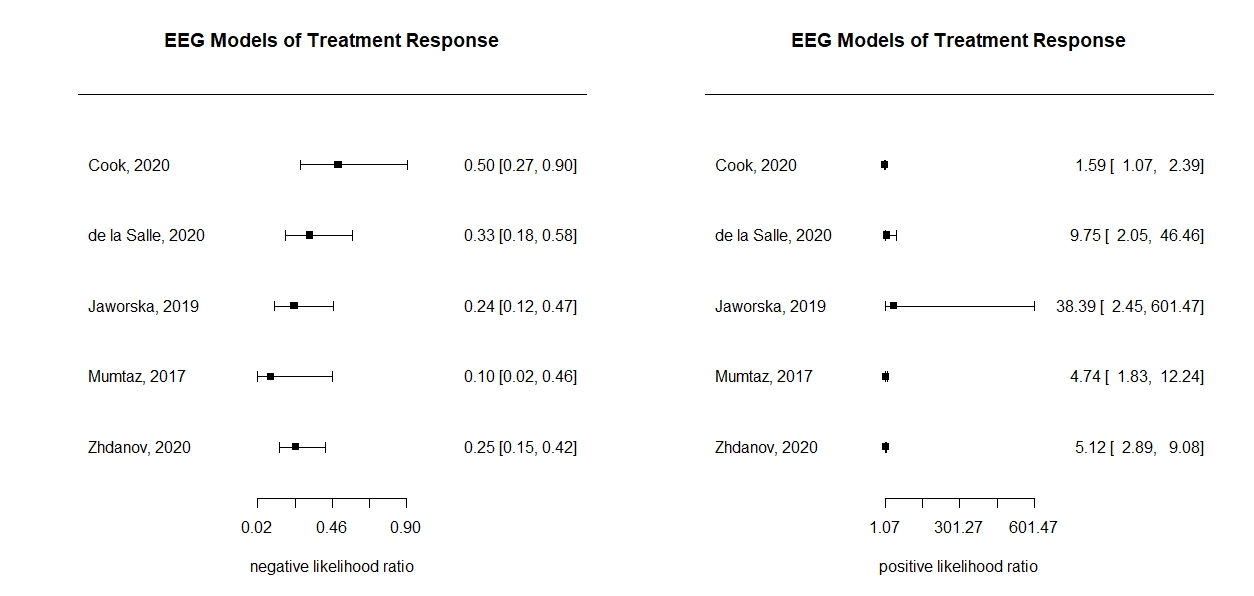


**Supplementary Figure S8: Negative and positive likelihood ratios of antidepressant treatment response prediction using EEG**

A depiction of the negative and positive likelihood ratios across studies, alongside 95% confidence intervals. Overall, the diagnostic odds ratio (DOR) across studies was 19.02 (95% CI: 5.51-65.61), with a positive likelihood ratio of 4.30 (95% CI: 1.92-9.64) and negative likelihood ratio of 0.296 (95% CI: 0.208-0.422). Of note, a large upper and lower bounds of the 95% confidence interval was observed within the DOR.
