# Supplementary material for: Predicting treatment response using EEG in major depressive disorder: A machine-learning meta-analysis
Source: Transl Psychiatry. 2022 Aug 12;12:332. doi: 10.1038/s41398-022-02064-z (PMC9374666; doi:10.1038/s41398-022-02064-z)
Supplement: Supplementary file 2 — Supplementary Tables [file 41398_2022_2064_MOESM2_ESM.docx]

**Supplementary Table S1 – Machine learning studies predicting treatment response using EEG in Major Depressive Disorder** (excluded studies)

| **First author, year** | **Sample size and diagnosis ^1,2^** | **Intervention** | **Outcome** | **Machine learning model** | **Accuracy** | **Other measures** |
| --- | --- | --- | --- | --- | --- | --- |
| **STUDIES PREDICTING RESPONSE TO NEUROSTIMULATION THERAPY** | | | | | | |
| **Al-Kyasi, 2016** | 10 patients with MDD | 15 sessions of tDCS over 3 weeks | Responders vs. Non-responders  Responders defined as ≥ 50% decrease in MADRS scores after session 15 or 23 of tDCS | SVM  ELM  LDA | 76%  *Performance was averaged across all algorithms* | N/A |
| **Zandvakili, 2019** | 29 patients with comorbid MDD and PTSD | 33 sessions of 5 Hz left DLPFC rTMS | Responders vs. Non-responders  Responders defined as ≥50% decrease in IDS-SR scores from baseline to end of treatment | LASSO  SVM | LASSO  73-80.5%  SVM  74-78.6% | MDD  AUC: 0.83  Sensitivity: 47-94%  Specificity: 0-83%  PTSD:  AUC: 0.71  Sensitivity: 37-100%  Specificity: 0-100%  *Sensitivity and specificity of SVM model not reported* |
| **STUDIES PREDICTING RESPONSE TO PHARMACOLOGICAL TREATMENT** | | | | | | |
| **Khodayari-Rostamabad, 2013** | 22 patients with MDD | Open-label trial of SSRI antidepressant | Responders vs. Non-responders  Responders defined as ≥30% improvement between the pre- and post-treatment HAMD-17 scores. | MFA | 87.9% | Sensitivity: 94.9%  Specificity: 80.9% |
| **Rabinoff, 2011** | 25 patients with MDD | 8-week double-blinded trial of either: 1) fluoxetine, 2) venlafaxine or 3) placebo | Responder vs Non-responder  Responders defined as post-treatment HAM-D scores ≤10 points | CART | *Venlafaxine*  Balanced Accuracy: 91.5%  *Fluoxetine*  Balanced Accuracy: 85.5% | *Venlafaxine*  Sensitivity: 83%  Specificity: 100% PPV: 100%  NPV: 86%  *Fluoxetine*  Sensitivity: 71%  Specificity: 100% PPV: 100%  NPV: 75% |
| **Shahabi, 2021** | 30 patients with MDD | 4-week course of an SSRI | Responders vs Non-responders  Responders defined as ≥50% improvement in BDI-II scores from baseline to post-treatment | CNN | 95.74% | Sensitivity: 95.56%  Specificity: 95.64% |

*BDI,* Beck Depression Inventory; *CNN,* Convolutional Neural Network; DLPFC, Dorsolateral Prefrontal Cortex; *ELM,* Extreme Learning Machine; *GBM,* Gradient Boosting Machine, *HAM-D,* Hamilton Depression Rating Scale; *IDS-SR,* Inventory of Depressive Symptomatology (Self-Report); *kNN*, k-Nearest Neighbors; *LASSO*, least absolute shrinkage and selection operator; *LDA,* Linear Discriminant Analysis; *LR*, Logistic Regression; *MADRS*, Montgomery-Asberg Depression Rating Scale; *MFA,* Mixture of Factor Analysis; *PARZEN*, Parzen density estimation; *RF*, Random Forest; *SVM*, Support Vector Machine

**Supplementary Table S2 – Characteristics of EEG Systems**

| **First author, year** | **EEG System** | **Reference Choice** | **Impedance** | **Filtering Method** | **Electrooculogram used?** | **Electrocardiogram used?** | **Eyes Open (EO) Eyes Closed (EC)** |
| --- | --- | --- | --- | --- | --- | --- | --- |
| **STUDIES PREDICTING RESPONSE TO NEUROSTIMULATION THERAPY** | | | | | | |  |
| **Bailey, 2017** | 30-channel Ag/AgCl electrode EasyCap EEG system | CPz | <5 kΩ | Bandpass filter (1-80 Hz)  Band-stop filter (47-53 Hz) | No | No | EC |
| **Bailey, 2018** | 30-channel Ag/AgCl electrode EasyCap EEG system | CPz | <5 kΩ | Bandpass filter (1-80 Hz)  Band-stop filter (47-53 Hz) | No | No | EO/EC |
| **Corlier, 2019** | 64-channel ANT Neuro TMS-compatible EEG system | CPz | <10 kΩ | Bandpass filter (0.5-55 Hz) | Yes | No | NA |
| **Erguzel, 2014** | 19-channel Scan LT EEG amplifier and electrode cap (6 channels were used) | Linked Ears  M1 + M2, LE, RE | NA | Bandpass filter (0.15-30 Hz) | No | No | EC |
| **Erguzel, 2015** | 19-channel Scan LT EEG amplifier and electrode cap | Linked Ears  M1 + M2, LE, RE | NA | Bandpass filter (0.15-30 Hz) | No | No | EC |
| **Erguzel, 2016** | 19-channel Scan LT EEG amplifier and electrode cap | Linked Ears  M1 + M2, LE, RE | NA | Bandpass filter (0.15-30 Hz) | No | No | EC |
| **Hasanzadeh, 2019** | Mitsar-EEG 201  18 Ag/AgCL electrodes | Linked Ears  M1 + M2, LE, RE | NA | Bandpass filter (1-42 Hz) | No | No | EC |
| **STUDIES PREDICTING RESPONSE TO PHARMACOLOGICAL TREATMENT** | | | | | | | |
| **Cao, 2019** | Mindo-4S Jellyfish  4 dry electrodes (Fp1, Fp2, AF7, AF8) | A2 | NA | Bandpass filter (1-12 Hz) | No | No | EC |
| **Cook, 2020** | Covidien BIS Complete 4-Channel Monitor  4 channel system (FPz, FT7, FT8, A1/A2) | A1+A2 | NA | NA | No | No | NA |
| **De la Salle, 2020** | 32 channel EasyCap EEG with Ag/AgCl electrodes | Common Average Reference | ≤5 kΩ | Bandpass filter (0.1-30 Hz) | Yes | No | EC |
| **Jaworska, 2019** | 32 channel EasyCap EEG with Ag/AgCl electrodes | Common Average Reference | ≤5 kΩ | Bandpass filter (0.1-30 Hz) | Yes | No | EC |
| **Mumtaz, 2017** | 19 channel electro-gel sensors with linked ear references - Brain Master Discovery amplifier was used | Linked Ear Reference | NA | Bandpass filter (0.1-70 Hz) | No | No | EC/EO |
| **Rajpurkar, 2020** | Scan LT EEG amplifier and electrode cap  6 frontal electrodes used *(Fp1, Fp2, F3, F4, F7, an F8)* | NA | NA | NA | No | No | EC |
| **Wu, 2020** | *Data from four studies*  BioSemi (72 channels)  NeuroScan Synamp (62 channels)  NeuroScan Synamp (60 channels)  Geodesic Net (129 channels) | Common Average Reference | <50 kΩ | 0.01 Hz high-pass filter  100 Hz low-pass filter | No | No | EC/EO |
| **Zhdanov, 2020** | *Data from four sites*  58 electrodes | Common Average Reference | NA | Bandpass filter (1 - 80 Hz)  Notch-filtered at 60 Hz | No | No | EC |
|  | | | | | | | |

**Supplementary Table S3 – Quality Assessment of Diagnostic Accuracy Studies-2 (QUADRS-2)**

| **Study** | **RISK OF BIAS** | | | | **APPLICABILITY CONCERNS** | | |
| --- | --- | --- | --- | --- | --- | --- | --- |
|  | **PATIENT SELECTION** | **INDEX TEST** | **REFERENCE STANDARD** | **FLOW AND TIMING** | **PATIENT SELECTION** | **INDEX TEST** | **REFERENCE STANDARD** |
| Bailey, 2017 | ☺ | ☺ | ☹ | ☺ | ☺ | ☺ | ☺ |
| Bailey, 2018 | ? | ☺ | ☹ | ☺ | ☺ | ☺ | ☺ |
| Cao, 2019 | ☹ | ☺ | ☺ | ☺ | ☺ | ☺ | ☺ |
| Cook, 2020 | ☺ | ? | ? | ☺ | ☺ | ☺ | ☺ |
| Corlier, 2019 | ☺ | ☺ | ☹ | ☺ | ☺ | ☺ | ☺ |
| De la Salle, 2020 | ☺ | ? | ☹ | ☺ | ☺ | ☺ | ☺ |
| Erguzel, 2014 | ☺ | ☺ | ☹ | ☺ | ☺ | ☺ | ☺ |
| Erguzel, 2015 | ☹ | ? | ☹ | ☺ | ☺ | ☺ | ☺ |
| Erguzel, 2016 | ☺ | ☺ | ☹ | ☺ | ☺ | ☺ | ☺ |
| Hasanzadeh, 2019 | ☺ | ? | ☹ | ☺ | ☺ | ☺ | ☺ |
| Jaworska, 2019 | ☺ | ☺ | ☺ | ☺ | ☺ | ☺ | ☺ |
| Mumtaz, 2017 | ☺ | ☺ | ☹ | ☺ | ? | ☺ | ☺ |
| Rajpurkar, 2020 | ☺ | ☺ | ☺ | ☺ | ☺ | ☺ | ☺ |
| Wu, 2020 | ☺ | ☺ | ☺ | ☺ | ☺ | ☺ | ☺ |
| Zhdanov, 2020 | ☺ | ☺ | ☺ | ☺ | ☺ | ☺ | ☺ |

☺Low Risk ☹High Risk ? Unclear Risk

| **Authors** | **Classification Task** | **Method to address class imbalance** | **True and False Positive/Negative** | **Performance Metrics** | **95% Confidence Intervals of Accuracy** |
| --- | --- | --- | --- | --- | --- |
| Bailey, 2017 | Responders  (≥ 50% improvement in HAMD-17)  vs  *Non-responders* (10/29) | N/A | TP = 9  FP = 1  TN = 26  FN = 3 | Balanced Accuracy = 91%  Sensitivity = 90%  Specificity = 92%  False Positive = 8%  False Negative = 10%  Standard Error = 5.20  Standard Deviation = 32.47 | Accuracy = 91%  (95% CI: 77.36-97.76) |
| Bailey, 2018 | *Responders*  (> 50% improvement in HAMD-17)  vs  *Non-responders* (12/30) | Class weights | TP = 10  FP = 2  TN = 26  FN = 4 | Balanced Accuracy = 86.50%  Sensitivity = 84%  Specificity = 89%  False Positive = 11%  False Negative = 16%  Standard Error = 2.27  Standard Deviation = 12.84 | Accuracy = 86.60%  (95% CI: 82.14-91.06) |
| Corlier, 2019 | *Responders*  (≥ 49% improvement in IDS-30)  vs  *Non-responders* (68/41) | N/A | TP = 45  FP = 12  TN = 22  FN = 29 | Balanced Accuracy = 69%  Sensitivity = 67.1% (19.2)  Specificity = 70.9% (13.3)  False Positive = 29.1%  False Negative = 32.9%  Standard Error = 4.65  Standard Deviation = 48.54 | Accuracy = 68.50%  (95% CI: 58.86-77.10) |
| Erguzel, 2014 | *Responders*  (≥50% improvement in HAMD-17)  vs  *Non-responders* (90/57) | N/A | Not available | Balanced Accuracy = N/A  Sensitivity = 84.44%  Specificity = N/A  False Positive = N/A  False Negative = 15.56%  Standard Error = N/A Standard Deviation = N/A | Accuracy = 80.25% |
| Erguzel, 2015 | *Responders*  (≥50% improvement in HAMD-17)  vs  *Non-responders*  (30/25) | N/A | TP = 28  FP = 4  TN = 21  FN = 2 | Balanced Accuracy = 88.66%  Sensitivity = 93.33%  Specificity = 84.00%  False Positive = 16%  False Negative = 6.7%  Standard Error = 4.61 Standard Deviation = 34.18 | Accuracy = 89.09%  (95% CI: 77.85-95.94) |
| Erguzel, 2016 | *Responders*  (≥50% improvement in HAMD-17)  vs  *Non-responders*  (90/57) | N/A | TP = 76  FP = 5  TN = 52  FN = 14 | Balanced Accuracy = 87.70%  Sensitivity = 84.30%  Specificity = 91.11%  False Positive = 8.8%  False Negative = 15.7%  Standard Error = 2.92  Standard Deviation = 35.40 | Accuracy = 86.4%  (95% CI: 80.56-92.04) |
| Hasanzadeh, 2019 | *Responders*  (≥50% improvement in HAMD-24)  vs  *Non-responders*  (23/23) | N/A | TP = 20  FP = 1  TN = 22  FN = 3 | Balanced Accuracy = 91.3%  Sensitivity = 87%  Specificity = 95.7%  False Positive = 4.3%  False Negative = 13%  Standard Error = 4.68  Standard Deviation = 31.74 | Accuracy = 91.3%  (95% CI: 79.21-97.58) |
| Cao, 2019 | *Responders*  (≥45% improvement in HAMD-17)  vs  *Non-responders*  (16/21) | Oversampling minority class | TP = 13  FP = 2  TN = 19  FN = 3 | Balanced Accuracy = 87%  Sensitivity = 82.1%  Specificity = 91.9%  False Positive = 8.1%  False Negative = 17.9%  Standard Error = 6.18  Standard Deviation = 37.59 | Accuracy = 81.3%  (95% CI: 71.23-95.47) |
| Cook, 2020 | *Remission*  (≤7 HAMD-17)  vs  *Non-remission* (38/35) | N/A | TP = 28  FP = 16  TN = 19  FN = 10 | Balanced Accuracy = 64.8%  Sensitivity = 74.3%  Specificity = 55.3%  False Positive = 44.7%  False Negative = 25.7%  Standard Error = 5.85 Standard Deviation = 49.98 | Accuracy = 64.4%  (95% CI: 52.30-75.24) |
| Salle, 2020 | *Responders*  (≥50% improvement in MADRS)  vs  *Non-responders* (27/20) | N/A | TP = 19  FP = 1  TN = 19  FN = 8 | Balanced Accuracy = 82.5%  Sensitivity = 70%  Specificity = 95%  False Positive = 5%  False Negative = 30%  Standard Error = 6.13 Standard Deviation = 42.02 | Accuracy = 80.96%  (95% CI: (66.87-90.93) |
| Jaworska, 2019 | *Responders*  (≥50% improvement in MADRS)  vs  *Non-responders*  (27/24) | N/A | TP = 21  FP = 0  TN = 24  FN = 6 | Balanced Accuracy = 88%  Sensitivity = 77%  Specificity = 99%  PPV = 99%  NPV = 81%  Standard Error = 4.95  Standard Deviation = 35.35 | Accuracy = 88.24%  (95% CI: 76.14-95.56) |
| Mumtaz, 2017 | *Responders*  ≥50% improvement in BDI-II)  vs  *Non-responders*  (17/17) | N/A | TP = 17  FP = 4  TN = 14  FN = 1 | Balanced Accuracy = 87.5%  Sensitivity = 95%  Specificity = 80%  False Positive = 20%  False Negative = 5%  Standard Error = 6.33  Standard Deviation = 37.44 | Accuracy = 86.11%  (95% CI: 70.50-95.33) |
| Zhdanov, 2020 | *Responders*  (≥50% improvement in MADRS)  vs  *Non-responders*  (55/67) | N/A | *Model 1*  TP = 43  FP = 10  TN = 57  FN = 11  *Model 2*  TP = 37  FP = 6  TN = 61 FN = 18 | Balanced Accuracy = 79.2%  Sensitivity = 67.3%  Specificity = 91.0%  False Positive = 9%  False Negative = 32.7%  Standard Error = 3.78  Standard Deviation = 41.75  Balanced Accuracy = 82.35%  Sensitivity = 79.2%  Specificity = 85.5%  False Positive = 14.5%  False Negative = 20.8%  Standard Error = 3.63  Standard Deviation = 40.09 | Accuracy = 80.33%  (95% CI: 72.12-86.97)  Accuracy = 82.4%  (95% CI: 74.68-88.91) |

**Supplementary Table S4: Confusion Matrices of Classification Models**

False positive rate is calculated as 1-specificity, while false negative is calculated as 1-sensitivity. In cases where confidence intervals were not reported, this metric was calculated using the true/false positive/negative ratios, as well as the prevalence of the positive class (responders). Standard error was imputed by subtracting the upper bound of the 95% CI from the lower bound and dividing by 3.92 (upper bound - lower bound)/3.92. Additionally, confusion matrices were provided according to the method used to address class imbalance, where applicable. It is important to note that none of the included studies reported the true positives/true negatives and false positives/false negative rates, and the numbers indicated in the table reflect calculations based on the prevalence, sensitivity, specificity, and total sample size. Summary statistics that were not reported in studies are indicated as N/A.

**Supplementary Table S5: Resting-state and task-specific EEG**

| **First author, year** | Resting state EEG used? | Task-specific EEG used? | Comments |
| --- | --- | --- | --- |
| **STUDIES PREDICTING RESPONSE TO NEUROSTIMULATION THERAPY** | | | |
| **Bailey, 2017** | No | Yes | Sternberg Working Memory Task |
| **Bailey, 2018** | Yes | No |  |
| **Corlier, 2019** | Yes | No |  |
| **Erguzel, 2014** | Yes | No |  |
| **Erguzel, 2015** | Yes | No |  |
| **Erguzel, 2016** | Yes | No |  |
| **Hasanzadeh, 2019** | Yes | No |  |
| **STUDIES PREDICTING RESPONSE TO PHARMACOLOGICAL TREATMENT** | | | |
| **Cao, 2019** | Yes | No |  |
| **Cook, 2020** | Yes | No |  |
| **De la Salle, 2020** | Yes | No |  |
| **Jaworska, 2019** | Yes | No |  |
| **Mumtaz, 2017** | No | Yes | 3-stimulus visual Oddball Task |
| **Rajpurkar, 2020** | Yes | No |  |
| **Wu, 2020** | Yes | No |  |
| **Zhdanov 2020** | Yes | No |  |
|  | | | |
