# Supplementary material for: Predicting treatment response using EEG in major depressive disorder: A machine-learning meta-analysis
Source: Transl Psychiatry. 2022 Aug 12;12:332. doi: 10.1038/s41398-022-02064-z (PMC9374666; doi:10.1038/s41398-022-02064-z)
Supplement: Supplementary file 3 — Supplementary Material [file 41398_2022_2064_MOESM3_ESM.docx]

ML Quality Scores of All Studies

| *Predicting response to Neurostimulation* | | | | | | | | | | |
| --- | --- | --- | --- | --- | --- | --- | --- | --- | --- | --- |
| Authors | Representative | Confounding | Outcome | ML | Feature Selection | Class imbalance | Missing data | Performance | Testing/  Validation | Overall Score |
| Bailey, 2017 | No | Yes | Yes | Yes | No | No | Yes | Yes | No | 5/9 |
| Bailey, 2018 | No | Yes | Yes | Yes | No | No | Yes | Yes | No | 5/9 |
| Corlier, 2019 | No | Yes | Yes | Yes | Yes | No | Yes | Yes | Yes | 7/9 |
| Erguzel, 2014 | No | No | Yes | Yes | Yes | No | Yes | Yes | No | 5/9 |
| Erguzel, 2015 | No | No | Yes | Yes | Yes | No | Yes | Yes | No | 5/9 |
| Erugzel, 2016 | No | No | Yes | Yes | Yes | No | Yes | Yes | No | 5/9 |
| Hasanzadeh, 2019 | No | Yes | Yes | Yes | Yes | No | Yes | Yes | No | 6/9 |
|  | | | | | | | | | | |

ML Quality Scores of All Studies

| *Predicting treatment response to psychiatric medication* | | | | | | | | | | |
| --- | --- | --- | --- | --- | --- | --- | --- | --- | --- | --- |
| Authors | Representative | Confounding | Outcome | ML | Feature Selection | Class imbalance | Missing data | Performance | Testing/  Validation | Overall Score |
| Cao, 2019 | No | Yes | Yes | Yes | Yes | No | Yes | Yes | Yes | 7/9 |
| Cook, 2020 | No | Yes | Yes | Yes | No | No | Yes | Yes | No | 5/9 |
| Jaworska, 2019 | No | Yes | Yes | Yes | Yes | No | Yes | No | No | 5/9 |
| Rajpurkar, 2020 | Yes | Yes | Yes | Yes | Yes | No** | Yes | Yes | No | 7/9 |
| De la Salle, 2020 | No | Yes | Yes | Yes | No | No | Yes | Yes | No | 5/9 |
| Mumtaz, 2017 | No | Yes | Yes | Yes | Yes | No | Yes | Yes | No | 6/9 |
| Wu, 2020 | Yes | Yes | Yes | Yes | Yes | No** | Yes | Yes | Yes | 9/9 |
| Zhdanov, 2020 | Yes | Yes | Yes | Yes | Yes | No | Yes | Yes | Yes | 8/9 |
| ** Class imbalance methods are not applicable to regression-based models | | | | | | | | | | |

**2. Quality assessment instrument development**

We formed a group of multidisciplinary researchers from the fields of Neuroscience, Psychiatry, and Computer Science to develop a time efficient and practical assessment strategy to evaluate the quality of supervised machine learning based healthcare research. For that purpose, we attempted to capture the reliability of the results presented in each study and identify practical ways that methodology may be improved. This instrument is not intended to provide an exhaustive evaluation of all components of supervised machine learning studies, but rather provide a brief overview of common considerations in supervised models, including patient sample, the specific outcome, algorithm selection, and how performance was evaluated. In total, this comprised nine methodological features, including sample representativeness, confounding variables, and outcome assessments Relevant considerations of each methodological feature are discussed in further detail in the next sections. The six remaining dimensions assess the quality and specific components of the machine learning approach that were used in each study. In summary, this entails the algorithm or framework used, evidence that hyper-parameter optimization and feature selection procedures were used, whether authors provided details on how missing data and class imbalance problems were handled, the accuracy of a given model, and finally whether the model performance was tested in unseen data. These dimensions were qualitatively evaluated according to the information in section 3.

**3. Quality assessment instrument domains**

| **Methodological Feature** | **Considerations** |
| --- | --- |
| 1. Representativeness of the sample | Was the study representative of the heterogeneity observed in the target population? If not, was this related to the sampling method, insufficient sample size or inclusion/exclusion criteria? |
| 2. Confounding variables | Did the study control for the most relevant confounding variables? If so, were covariates assessed using subjective or objective measures? |
| 3. Outcome assessment | How were outcome measures assessed?  A. Independent blind assessment (✓)  B. Secure record (e.g., surgical records) (✓)  C. Interview not blinded, self-report or medical record  D. No description  *A-C scored as “Yes”; D scored as “No”* |
| 4. Machine learning approach | Was the machine learning algorithm used to analyse the data clearly described and appropriate? |
| 5. Feature selection | Did the study describe both feature selection and hyperparameter tuning? Which metrics were used? |
| 6. Class imbalance | Did the authors address the class imbalance problem? Which method was used? |
| 7. Missing data | Did the study describe how the authors handled missing data, including whether they were inputted or removed? |
| 8. Performance/accuracy | Were the following performance metrics included for classification studies?   1. Accuracy 2. Sensitivity 3. Specificity 4. AUC 5. PPV/NPV   Or, alternatively, were one of the following performance metrics included for regression studies?   1. Mean-squared error 2. Mean-absolute error 3. Root-mean-squared error |
| 9. Testing/validation | Was the test dataset "unseen" during model training? Was the model tested on a hold-out or an external dataset? |

*3.1. Representativeness of the sample*

Machine learning models can deal with large amounts of data and the problem of heterogeneity. Therefore, there is less of a need to be restrictive with inclusion and exclusion criteria, relative to a traditional statistical approach examining significant effects at a group-level. Considering all studies included in the present review used data from randomized clinical trials, determined whether 1) performance was tested on an external sample with differences in inclusion/exclusion criteria, and 2) whether a training sample of ≥ 100 patients was used in model development.

*3.2. Internal CV*

To adequately control for confounding variables within machine learning models, it is important to ensure that these variables have a similar effect across the entire sample. To achieve this, randomization is an important step within the analysis. Often, the overall sample is randomly split into training and testing sets, and the analysis is repeated on the training dataset with different hyperparameters to maximize accuracy and minimize error. This is known as internal cross-validation. From here, if model performance is similar in the testing dataset, it presumes that potential confounding variables are uniformly distributed across the sample. Using these criteria, we evaluated whether the authors controlled for confounding variables.

*3.3. Outcome assessment*

How an outcome is defined has several important implications in a predictive model. Depending on the question or problem, a classification task may be appropriate, which uses a categorical outcome, or a regression task may be more relevant, where the outcome is continuous and numeric. A clinical instrument or questionnaire, for example, can be used as a numeric score or it can be transformed into a categorical outcome by using a cut-off score. We evaluated how authors assessed these outcomes, considering (A) independent blind assessments and secure records as high quality, (B) unblinded interview, self-report or medical record as lower quality and (C) when no description was available.

*3.4. Algorithm selection*

There are several algorithms to choose from, with each relying on slightly different assumptions of the underlying data. Broadly speaking, there are linear (logistic regression, linear support vector machine), non-linear (Naive Bayes, K-Nearest Neighbors, Learning Vector Quantization)

tree-based (decision trees, random forest, xgboost) and neural network (convolutional neural network, multilayer perceptrons) models, although others exist. Certain algorithms may be better suited to problems. For example, tree-based models such as random forest may be better suited to datasets with multicollinearity among features than linear-based models such as logistic regression. However, regularization parameters can be used in linear-based models (such as L2 regularization) to account for issues such as this.

Nevertheless, it is often difficult to determine beforehand which algorithms will lead to the highest model performance. Therefore, it is often a good strategy to compare the model performance of several algorithms. In this item, we evaluated whether the authors used an algorithm that is commonly used for the specific type of dataset, if several algorithms were compared, and if hyperparameter tuning was used.

The appropriateness of a machine learning algorithm was determined based on whether the specific data used in model development was congruent or incongruent with the strengths and limitations of the specific algorithm. For example, if a Gaussian process model was used, which is a non-sparse algorithm that loses efficiency in high dimensional spaces, in conjunction with a high-dimensional dataset, this algorithm would be deemed inappropriate for the input data. Conversely, Naive Bayes, which works well with high dimensional data would be considered an appropriate algorithm in such cases. Another example of an inappropriate model would be the use of convolutional neural networks for structural and tabular style datasets, as such algorithms are better suited to unstructured datasets. In cases where authors included both appropriate and inappropriate algorithms during model development, this consideration is scored with a “B”, alongside an asterisk to indicate which algorithms were inappropriate and why. Studies which only utilized one algorithm during model development that was deemed inappropriate received a score of “C”. Furthermore, studies are scored with a “B” if they did not compare multiple algorithms during model development and were scored as an “A” if they compared multiple algorithms that were deemed appropriate based on the candidate feature set.

*3.5. Feature selection*

A common problem in machine learning studies is the so-called small-n-large-p problem, also known as the curse of dimensionality, which occurs when there are more variables than examples in a dataset. Machine learning models created using these datasets are more prone to overfitting, which often results in overinflated performance in a training dataset, but much poorer performance in an external testing dataset. In addition, some algorithms cannot deal with more dimensions than examples. Highly correlated variables can also introduce more importance to a specific characteristic, decreasing the importance of the remaining variables. To circumvent these issues, a proper feature selection procedure, when applicable, should be done prior to training or as part of the training procedure, such as it happens in embedded methods. The feature selection can be knowledge-driven or data-driven. In this item, we examined if the study used a proper feature selection (if applicable).

*3.6. Class imbalance*

Class imbalance occurs when the distribution of the outcome classes is highly unbalanced, i.e., when one outcome occurs much more frequently than the other outcome(s). This may result in a model with high accuracy but with very little clinical utility. For example, let us suppose that we have 95 occurrences of response in our dataset and only 5 occurrences of a nonresponse. Even if our model has 95% accuracy, it is useless if the model cannot detect the five instances of non-response high accuracy. In this item, we evaluated whether there was a class imbalance in the sample and if this problem was correctly addressed. This can be done using a series of methods, including (1) changing the metric of performance (accuracy, for example, is a poor form of evaluating imbalanced data sets; (2) resampling the data set by artificially increasing it (oversampling) or by removing examples from the majority class to create a more balanced data set (under-sampling); (3) by generating more data with algorithms such as the Synthetic Minority Over-Sampling Technique (SMOTE); (4) by choosing algorithms that deal better with unbalanced classes, such as CART or random forests; (5) by using penalized models; or (6) by using anomaly and change detection. In cases where class imbalance was not relevant (balanced classes or regression models) this is scored as “yes”.

*3.7. Missing data*

It is critical to handle missing data since several algorithms cannot process incomplete data sets. Furthermore, it is also necessary to use an adequate imputation method to avoid introducing bias, which would otherwise lead to false conclusions if not addressed. It is important to report the amount of missing data in each variable, if these cases were excluded, or if the authors used an algorithm to input data and which algorithm/technique was used. Ideally, authors should provide a visual distribution of the patterns of missing data, such as aggregation plots, spinogram/spineplots, mosaic plots, etc. All these factors were evaluated in this section.

*3.8. Performance/accuracy*

Here, we evaluate whether the authors reported all relevant results and if they used the appropriate metrics. Studies informing only partial metrics may mask bias and flaws of the method, preventing the reader from fully understanding the relevance of the model. Confidence intervals should ideally be available for all performance metrics.

*3.9. Testing/Validation*

We can divide the machine learning process into three main components: training, validation, and testing. A training set allows the algorithm to learn and develop a predictive model. The validation set contains unseen data and is used to control for overfitting. Frequently, the same dataset is divided into training and validation sets. After a model is trained and validated, and shows consistent performance in both these steps, the model can be applied in an external and independent testing set. This allows us to see if the model can be generalized outside of the original sample. Some validation methods include holdout validation, k-fold, and leave one out cross validation.

A model that shows good performance in the training set but performs significantly poorer in the validation step is most likely due to overfitting - which occurs when the model relies more on the specific nuances and noise of the training dataset, resulting in poor accuracy in unseen data. In this item, we evaluated whether the authors properly tested and validated their models by taking steps to improve its generalizability. It is important to highlight that the use of cross-validation to evaluate performance should be discouraged when the data is large enough for a training-test split. Furthermore, the size of the test set should be sufficiently large for accuracy and other metrics to be estimated with high reliability.

4. Search Filter

**PubMed/MEDLINE**

Abbreviated Search: (“Supervised Machine Learning” OR “Artificial intelligence”) AND (“Major Depressive Disorder”) AND (“Electroencephalography”) AND (“Intervention” OR “Treatment”)

Full Search:

((((((((((((((((((((((((((((((((((((((((((Artificial Intelligence[MeSH Major Topic]) OR (Supervised Machine Learning[MeSH Major Topic])) AND (Depressive Disorder, Major[MeSH Major Topic])) OR (Major Depressive Disorders[MeSH Terms])) OR (Major Depressive Disorder[MeSH Terms])) OR (Depressive Disorders[MeSH Terms])) OR (Neurosis, Depressive[MeSH Terms])) OR (Depressive Neuroses[MeSH Terms])) OR (Depressive Neurosis[MeSH Terms])) OR (Neuroses, Depressive[MeSH Terms])) OR (Depression, Endogenous[MeSH Terms])) OR (Depressions, Endogenous[MeSH Terms])) OR (Endogenous Depression[MeSH Terms])) OR (Endogenous Depressions[MeSH Terms])) OR (Depressive Syndrome[MeSH Terms])) OR (Depressive Syndromes[MeSH Terms])) OR (Syndrome, Depressive[MeSH Terms])) OR (Syndromes, Depressive[MeSH Terms])) OR (Depression, Neurotic[MeSH Terms])) OR (Depressions, Neurotic[MeSH Terms])) OR (Neurotic Depression[MeSH Terms])) OR (Neurotic Depressions[MeSH Terms])) OR (Melancholia[MeSH Terms])) OR (Melancholias[MeSH Terms])) OR (Unipolar Depression[MeSH Terms])) OR (Depression, Unipolar[MeSH Terms])) OR (Depressions, Unipolar[MeSH Terms])) OR (Dysthmic Disorder[MeSH Terms])) OR (Disorder, Dysthymic[MeSH Terms])) OR (Dysthymic Disorders[MeSH Terms])) OR (Dysthymia[MeSH Terms])) OR (Persistent Depressive Disorder, Dysthymia[MeSH Terms])) OR (Dysthymia and Chronic Depression[MeSH Terms])) OR (Neurotic Depression, Persistent Depressive Disorder[MeSH Terms])) AND (Electroencephalography[MeSH Major Topic])) OR (EEG[MeSH Terms])) OR (Electroencephalogram[MeSH Terms])) OR (Electroencephalograms[MeSH Terms])) OR (Brain Waves[MeSH Major Topic])) AND (Clinical Trials as Topic[MeSH Major Topic])) OR (Treatment response[Other Term])) OR (treatment prediction[Other Term])) OR (treatment selection[Other Term]))

Date: 2022-02-11

Retrieved references: 1827

**Scopus**

Abbreviated Search: (Supervised Machine Learning OR Artificial Intelligence) AND (Major Depressive Disorder) AND (Electroencephalography) AND (Intervention OR Treatment)

Full Search: ((Artificial Intelligence) OR (Supervised machine Learning)) AND ((Depressive Disorder, Major) OR (Major Depressive Disorders) OR (Depressive Disorders) OR (Neurosis, Depressive) OR (Depressive Neurosis) OR (Neuroses, Depressive) OR (Depression, Endogenous) OR (Depressions, Endogenous) OR (Endogenous Depression) OR (Endogenous Depressions) OR (Depressive Syndrome) OR (Depressive Syndromes) OR (Syndrome, Depressive) OR (Syndromes, Depressive) OR (Depression, Neurotic) OR (Depressions, Neurotic) OR (Neurotic Depression) OR (Neurotic Depressions) OR (Melancholia) OR (Melancholias) OR (Unipolar Depression) OR (Depression, Unipolar) OR (Depressions, Unipolar) OR (Unipolar Depressions) OR (Dysthmic Disorder) OR (Disorder, Dysthymic) OR (Dysthymic Disorders) OR (Dysthymia) OR (Persistent Depressive Disorder, Dysthymia) OR (Dysthymia and Chronic Depression) OR (Neurotic Depression, Persistent Depressive Disorder)) AND ((Electroencephalography) OR (EEG) OR (Electroencephalogram) OR (Electroencephalograms) OR (Brain Waves)) AND (Clinical Trials) OR (Treatment Response) OR (Treatment Prediction) OR (Treatment Selection)

Date: 2022-02-11

Retrieved References: 1466

**Web of Science**

Search: (TS= Algorithms OR Machine Learning OR Artificial Intelligence) AND (TS= Major Mental Disorder) AND (TS =Electroencephalography OR Magnetoencephalography) AND (TS = Intervention OR Treatment)

Full Search:

 (TS=(Artificial Intelligence) OR TS= (Machine Learning)) AND (TS=(Major Depressive Disorder) OR (TS=Depressive Disorder, Major) OR (TS=Major Depressive Disorders) OR (TS=Depressive Disorders) OR (TS=Depression) OR (TS=Dysthymia) OR (TS=Neurosis, Depressive) OR (TS=Depressive Neurosis) OR (TS=Neuroses, Depressive) OR (TS=Depression, Endogenous) OR (TS=Depressions, Endogenous) OR (TS=Endogenous Depression) OR (TS=Endogenous Depressions) OR (TS=Depressive Syndrome) OR (TS=Depressive Syndromes) OR (TS=Syndrome, Depressive) OR (TS=Syndromes, Depressive) OR (TS=Depression, Neurotic) OR (TS=Depressions, Neurotic) OR (TS=Neurotic Depression) OR (TS=Neurotic Depressions) OR (TS=Melancholia) OR (TS=melancholicas) OR (TS=Unipolar Depression) OR (TS=Depression, Unipolar) OR (TS=Depressions, Unipolar) OR (TS=Unipolar Depressions) OR (TS=Dysthmic Disorder) OR (TS=Disorder, Dysthymic) OR (TS=Dysthymic Disorders) OR (TS=Dysthymia) OR (TS=Persistent Depressive Disorder, Dysthymia) OR (TS=Dysthymia) OR (TS=Chronic Depression) OR (TS=Neurotic Depression, Persistent Depressive Disorder)) AND (TS=(Electroencephalography) OR (AB=EEG) OR (TS=Electroencephalogram) OR (TS=Electroencephalograms) OR (TS=Brain Waves)) AND (TS=(Clinical Trials) OR (TS=Treatment Response) OR (TS=Treatment Prediction) OR (TS=Treatment Selection) OR (TS=Treatment) OR (TS=Therapy))

Date: 2022-02-11

References Retrieved: 53

**DUPLICATES**

Retrieved References (without duplicates): 2489

Removed Duplicates: 857
